# Cascade anchoring strategy for general mass production of high-loading single-atomic metal-nitrogen catalysts

**DOI:** 10.1038/s41467-019-09290-y

**Published:** 2019-03-20

**Authors:** Lu Zhao, Yun Zhang, Lin-Bo Huang, Xiao-Zhi Liu, Qing-Hua Zhang, Chao He, Ze-Yuan Wu, Lin-Juan Zhang, Jinpeng Wu, Wanli Yang, Lin Gu, Jin-Song Hu, Li-Jun Wan

**Affiliations:** 1 Beijing National Laboratory for Molecular Sciences (BNLMS), CAS Key Laboratory of Molecular Nanostructure and Nanotechnology, Institute of Chemistry, Chinese Academy of Sciences, Beijing, 100190 China; 2 University of Chinese Academy of Sciences, Beijing, 100049 China; 3 College of Chemistry and Materials Science, Sichuan Normal University, Chengdu, 610068 China; 4 Beijing National Research Center for Condensed Matter Physics, Collaborative Innovation Center of Quantum Matter, Institute of Physics, Chinese Academy of Sciences, Beijing, 100190 China; 5 Shanghai Synchrotron Radiation Facility, Shanghai Institute of Applied Physics, Chinese Academy of Sciences, Shanghai, 201800 China; 6 Advanced Light Source, Lawrence Berkeley National Laboratory, Berkeley, California 94720 USA

## Abstract

Although single-atomically dispersed metal-N_x_ on carbon support (M-NC) has great potential in heterogeneous catalysis, the scalable synthesis of such single-atom catalysts (SACs) with high-loading metal-N_x_ is greatly challenging since the loading and single-atomic dispersion have to be balanced at high temperature for forming metal-N_x_. Herein, we develop a general cascade anchoring strategy for the mass production of a series of M-NC SACs with a metal loading up to 12.1 wt%. Systematic investigation reveals that the chelation of metal ions, physical isolation of chelate complex upon high loading, and the binding with N-species at elevated temperature are essential to achieving high-loading M-NC SACs. As a demonstration, high-loading Fe-NC SAC shows superior electrocatalytic performance for O_2_ reduction and Ni-NC SAC exhibits high electrocatalytic activity for CO_2_ reduction. The strategy paves a universal way to produce stable M-NC SAC with high-density metal-N_x_ sites for diverse high-performance applications.

## Abstract (web summary)

Although single atom catalysts (SACs) with high-loading metal-Nx have great potential in heterogeneous catalysis, their scalable synthesis remains challenging. Here, the authors develop a general cascade anchoring strategy for the mass production of a series of metal-Nx SACs with a metal loading up to 12.1 wt%.

## Introduction

Single-atom catalyst (SAC) has recently emerged as a rising-star in catalysis since it combines the merits of both heterogeneous and homogeneous catalysts while bridges the gap between them with unique features. Comparing with heterogeneous catalysts, SAC maximizes the atom utilization and has homogenous active sites with tunable electronic environments for highly catalytic activity or/and selectivity, while simultaneously holds improved stability and excellent recyclability in contrast to homogeneous catalysts^1–10^. In the past a couple of years, dozens of SACs have been therefore developed for thermocatalytic reaction (such as CO oxidation^11,12^, water–gas shift reaction^12,13^, and methane conversion^14^), photocatalytic reaction (photocatalytic H_2_ evolution^15^ and CO_2_ reduction^16^), electrocatalytic reaction (H_2_ evolution^17–19^, O_2_ reduction^20–24^, CO_2_ reduction^25^, and N_2_ reduction^26^), as well as organic electrosynthesis^27^. Controllable preparation of SAC, however, still remains challenging in view of the strong tendency of migration and aggregation of active atoms during either the fabrication or the subsequent application processes. To this end, supporting monodispersed atoms on appropriate support represents the most feasible and effective way to achieve SAC. Until now, several strategies such as using confinement effect, coordination effect, or chemical bonding have been reported to synthesize isolated metal sites over supports by (1) limiting the loading amount of active component; (2) boosting the interactions between metal atom and support; or (3) employing defect or void on support^28–32^.

Among SACs, atomic metal–N_x_ (M–N_x_) moieties anchored on carbon support (M–NC) have attracted particular interests especially in electrocatalysis, since nitrogen can not only effectively anchor and stabilize single-metal atom on carbon but also modulate the electronic structures of metal or carbon atom to optimize the adsorption/desorption of intermediates for enhancing catalytic performance^33–37^. Moreover, carbon supports are readily available for commercial use and highly electrically conductive for accelerating electron transfer during reactions. Such single-atomic M–NC SACs have demonstrated extraordinary promise in catalytic oxidation of benzene to phenol^38^, chemoselective hydrogenation of nitroarenes to produce azo compounds^39^, semihydrogenation of 1-hexyne^40^, etc. Huang and Duan et al.^41^ recently reported a two-step approach to the synthesis of well-defined atomic MN_4_C_4_ (M = Fe, Co, Ni) moieties embedded in graphene with a metal loading of ~0.05 at% as efficient electrocatalysts for oxygen evolution reaction.

The catalytic activity and turnover efficiency of a catalyst and thus the power/energy density of catalyst-based devices closely depend on the number of catalytic sites, besides its intrinsic activity. One of big challenges for SAC is the low concentration of single-atomic sites, since the loading and the aggregation of atoms have to be balanced. Especially at elevated temperature, metal atoms are getting easier to migrate and aggregate, causing more challenging to achieve M–NC SACs with high metal loading since the formation of M–N bonding usually needs high temperature (such as over 700 °C)^42–45^. Although the progress has been made on the synthesis of M–NC SACs, few reports can achieve the metal loading over 4 wt%. Another challenge is the mass production of M–NC SACs, which is essential to their practical applications. Most strategies for the synthesis of SAC need delicate control of the defects in supports and synthetic procedures to stabilize single atoms, whereas the mass production requires the commercially available low cost supports and scalable, manageable processing. It is therefore highly desirable to develop a method compatible with large-scale production for synthesizing M–NC SACs with high metal loadings.

**Fig. 1:**
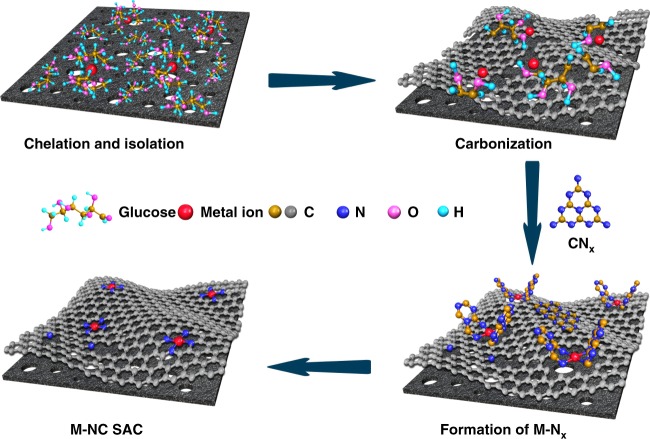
The cascade anchoring strategy for the synthesis of M–NC SACs. First, chelating agent (glucose) efficiently sequesters metal ions and binds to O-rich carbon support, while excessive glucoses physically isolate glucose–metal complexes on carbon substrate. Second, the chelated metal complexes further secure metal atoms via the decomposed residues up to certain temperature. Third, CN_x_ species decomposed from melamine at higher temperature subsequently capture metal atoms to form M–N_x_ moieties and integrate into the pyrolyzed carbon layer

Herein, we report a general approach to synthesize a wide range of M–NC SACs (M = Mn, Fe, Co, Ni, Cu, Mo, Pt, etc.) with metal loadings up to 12.1 wt% via a cascade anchoring strategy. As shown in Fig. 1, the metal ions are first chelated by chelating agent such as glucose here, then anchored onto oxygen-species-rich porous carbon support with high surface area (Step 1). The chelating agent can effectively sequester metal ions (primary protection) and bind to O-rich carbon support via the interaction with O-containing groups. The excessive chelating agent bound to support surface will physically isolate the metal complex (secondary protection). The complex bound carbon is then mixed with melamine as a nitrogen source for subsequent pyrolysis to achieve M–NC SACs. During pyrolysis, the chelated metal complex can further secure metal atoms via the decomposed residues up to certain temperature (~500 °C, Step 2) (tertiary protection), while carbon nitrogen species (CN_x_) (such as C_3_N_4_ etc.) decomposed from melamine at higher temperature (>~600 °C) can subsequently bind with metal atoms to form M–N_x_ moieties (Step 3), taking over the protection and preventing metal atoms from aggregation (quaternary protection). Systematical experiments reveal that such sequential protecting strategy allows for producing wide-ranging M–NC SACs with a high metal loading up to 12.1 wt%, since the chelating interaction can take place between a wide range of metal ions and ligands^46,47^. Moreover, the carbon support can be low-cost commercial porous carbon; the chelating agents can be low-cost carbohydrates, such as glucose etc.; and the processing is very easy to scale up. In this regard, the present strategy is suitable for the low-cost mass production of M–NC SACs for diverse applications. As a demonstration, Fe–NC SAC shows a superior electrocatalytic activity for oxygen reduction reaction (ORR) in 0.1 M KOH with a half-wave potential of 0.90 V (all potentials are versus to RHE) and a kinetic mass current of 100.7 A g^−1^ at 0.9 V, 50 mV and 65 A g^−1^ higher than that of state-of-the-art commercial Pt/C catalyst, respectively. Ni–NC SAC exhibits an excellent electrocatalytic activity for CO_2_ reduction to CO in terms of a good Faraday efficiency of 89% with a high current density of 30 mA cm^−2^ at −0.85 V.

## Results

### Synthesis and structural analysis of Fe–NC SAC

**Fig. 2:**
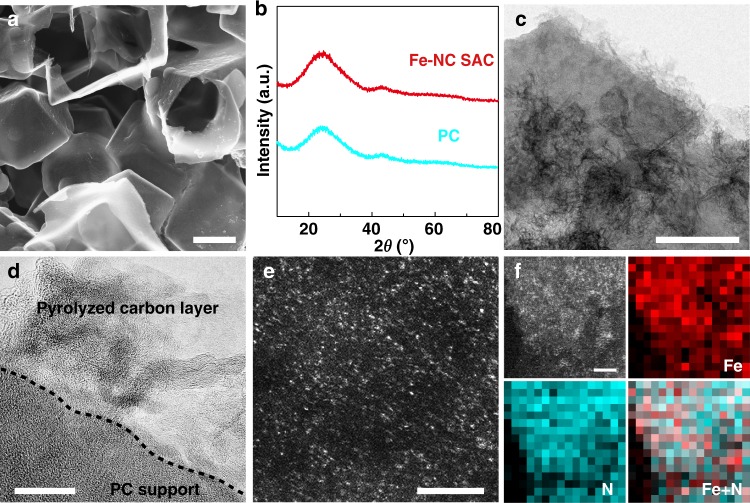
Structural characterizations of PC support and Fe–NC SAC. **a** SEM image of PC support. **b** XRD patterns of PC and Fe–NC SAC. **c** TEM, **d**, HRTEM, **e**, HAADF–STEM images of Fe–NC SAC. **f** HAADF–STEM image and EELS mapping images of Fe, N, and overlaid Fe and N on Fe–NC SAC. Scale bars, 1 μm (**a**); 200 nm (**c**); 20 nm (**d**); 3 nm (**e**); 2 nm (**f**)

Since Fe–NC electrocatalysts are particularly interested for ORR to replace precious Pt-based commercial catalysts in fuel cells etc. We first take Fe–NC SAC as an example to demonstrate our strategy and its application. Fe–NC SAC was prepared in two steps. First, porous carbon (PC) support was ultrasonically dispersed in the solution containing Fe source and glucose (chelating agent). Second, the dried powder was ground with melamine (nitrogen source), followed by pyrolysis at 800 °C to achieve Fe–NC SAC. The scanning electron microscope (SEM) image (Fig. 2a) shows that PC substrate (prepared by pyrolysis of potassium citrate as presented in the section of Methods) has a three-dimensional honeycomb-like morphology with plenty of macropores in several micrometers, which benefits mass transfer during both synthesis and application processes. X-ray diffraction (XRD) pattern (cyan curve in Fig. 2b) shows two typical broad peaks at 24.3 and 44.3° for the PC substrate. The specific surface area and pore volume are measured to be 1713 m^2^ g^−1^ and 0.75 cm^3^ g^−1^, respectively (Supplementary Fig. 1). The pore size distribution analysis indicates most of nanopores centered at 1.3 and 2.0 nm. X-ray photoelectron spectroscopy (XPS) spectrum reveals plenty of O-species (10.05 at%) on the surface of the PC substrate (Supplementary Fig. 2). X-ray energy dispersive spectroscopic (EDS)-mapping images show that the elemental O uniformly distributes on whole-carbon sheets (Supplementary Fig. 3). These features make such carbon substrate perfect for anchoring glucose-chelated Fe complex. The chelation of α-D-glucose and Fe(III) ions to form α-D-glucose–Fe(III) complex has been reported in the literatures^47,48^ and supported by our density functional theory (DFT) calculations (Supplementary Fig. 4). After mixing this PC substrate with glucose and Fe source, Fourier-transform infrared (FTIR) spectrum and EDS-mapping images indicate that glucose and glucose-chelated Fe complex cover on whole substrate in view of uniform FTIR signals of O–H and distribution of elemental Fe and O (Supplementary Figs. 5 and 6). After pyrolysis at 800 °C for 2 h, no additional XRD peaks are detected except for those from carbon (Fig. 2b), implying no formation of crystallized Fe. Transmission electron microscopy (TEM) and high-resolution TEM (HRTEM) images (Fig. 2c, d) show that carbon sheet support is covered by flocculent sheet-like structures, which is a typical feature of pyrolyzed carbonaceous materials (glucose). Raman spectra (Supplementary Fig. 7) indicate that the *I*_D_*/I*_G_ ratio for Fe–NC SAC is slightly higher than carbon substrate (1.3 vs. 1.2, PC substrate here went through the same pyrolysis for Raman recording), agreeing with the formation of the flocculent carbon layer in more disorder as observed in TEM image. No Fe-based particles, which are typically seen in pyrolyzed Fe–NC products, are found during TEM observation. XRD and TEM results suggest that Fe may exist in a form of single atom. For clearly revealing the state of Fe, the high-angle annular dark-filed scanning TEM (HAADF–STEM) was used to acquire the evidence of Fe distribution at atomic resolution. As displayed in Fig. 2e and Supplementary Fig. 8, a number of bright spots in a single-atom size are clearly observed, which can be safely attributed to Fe atoms in this sample. The average size of spots is 1.04 ± 0.35 Å on a basis of statistical analysis on over 400 bright spots (inset in Supplementary Fig. 8a), corroborating these Fe atoms are mainly in single-atomic state. EDS spectrum evidences the existence of elemental C, Fe, and N (Supplementary Fig. 9a). Electron energy loss spectroscopy (EELS) and EDS-mapping images depict the homogenous and uniform distribution of Fe and N in the Fe–NC SAC (Fig. 2f and Supplementary Figs. 9b–f and 10).

### Atomic structure analysis of Fe–NC SAC by XAFS and XPS

**Fig. 3:**
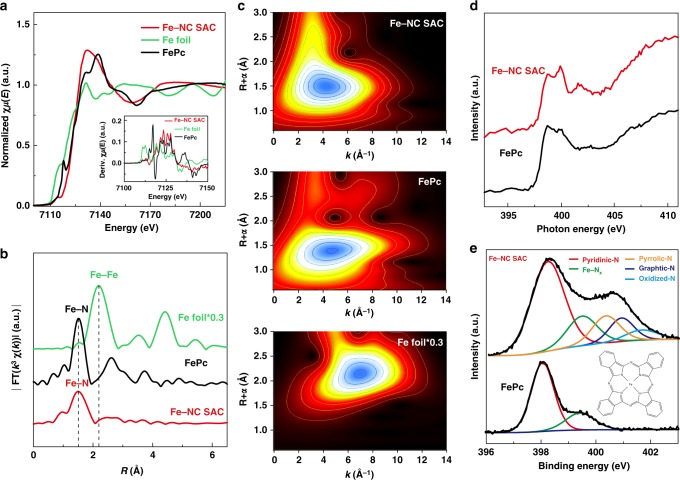
Atomic structure analysis of Fe–NC SAC by XAFS and XPS. **a** Fe K-edge XANES spectra (inset: first-derivative curves), **b** Fourier transform of Fe K-edge EXAFS spectra, and **c**, Wavelet transform of the *k*^3^-weighted EXAFS data of Fe–NC SAC and reference samples (FePc and Fe foil). **d** N *K*-edge NEXAFS spectra and **e**, deconvoluted N 1*s* XPS spectra of Fe–NC SAC and reference FePc

Element-selective X-ray absorption fine structure (XAFS) spectroscopy experiments were further conducted, including the extended X-ray absorption fine structure (EXAFS), which are powerful for determining the coordination environment and chemical state of absorbing centers with high sensitivity. As shown in Fig. 3a, X-ray absorption near-edge structure (XANES) spectrum and the first derivative XANES spectrum of Fe–NC SAC are very similar to those of the reference iron phthalocyanine (FePc) which has well-defined Fe–N_4_ coordinated sites^49–51^, while distinct from those of the metallic Fe foil. This means Fe state in Fe–NC SAC should be similar to that in FePc. Figure 3b shows the Fourier transform (FT) *k*^3^-weighted EXAFS spectra of Fe k-edge. Comparing with the Fe foil, no apparent peaks (2.20 and 4.42 Å) for Fe−Fe coordination are observed in Fe–NC SAC. As expected, FT EXAFS spectrum of Fe–NC SAC has a strong peak at 1.50 Å. In reference to FePc, this peak can be well assigned to Fe–N distance where a nitrogen shell surrounds one Fe atom. Wavelet transform (WT) was also used to investigate the Fe K-edge EXAFS oscillations of Fe–NC SAC and the references. As shown in Fig. 3c, WT analysis of Fe–NC SAC shows only one intensity maximum at about 4.5 Å^–1^ for Fe–NC SAC, which is very close to that in the reference FePc (~4.5 Å^–1^), but distinct from the feature of Fe foil (7.0 Å^–1^). Combining with above HAADF–STEM results that all Fe species are atomically dispersed without detectable aggregation, these analyses suggest that Fe in Fe–NC SAC exists in a similar state to the reference FePc. For giving further insights into the chemical configuration of Fe, FT EXAFS fittings in *R*, *q*, and *k* spaces were carried out to reveal the structural parameters and evaluate the fitting quality. As shown in Supplementary Figs. 11 and 12, all fittings are in good consistency with experimental data. The fitting results give an average coordination number of 4.3 for the first shell (Fe–N) and an average Fe–N bond length of 1.99 Å (see more details in Supplementary Table 1).

The chemical environments of N in Fe–NC SAC and reference FePc were further investigated by near-edge XAFS (NEXAFS) and XPS technique. The N K-edge NEXAFS spectrum of Fe–NC SAC shows three distinct peaks at about 398.8, 399.8, and 401.8 eV (Fig. 3d). The peak at 398.8 eV can be assigned to pyridinic state or the state similar to aza-bridge in FePc due to the same energy position. The peak at 399.8 eV shares the identical position with the Fe–N bonding in FePc, corroborating the existence of Fe–N bonding in Fe–NC SAC^52,53^. The wide peak at 401.8 eV could be assigned to pyrrolic or other nitrogen states according to the literatures^54,55^. For XPS spectra shown in Fig. 3e, the N 1*s* signal for Fe–NC SAC can be deconvoluted into several characteristic peaks. The XPS signals at 398.3, 399.5, 400.4, 400.9, and 401.7 eV are assigned to pyridinic-N, Fe–N_x_, pyrrolic-N, graphitic-N, and oxidized-N, respectively^56–58^. The peak at 399.5 eV indicates the presence of N in the chemical state similar to Fe–N_x_ moiety in FePc.^59,60^ The high-resolution Fe 2*p* XPS spectrum (Supplementary Fig. 13) indicates that Fe exists in form of iron (II). No clear signal of metallic Fe is detected. These XPS data agree with the existence of Fe–N_x_ coordination in Fe–NC SAC. Moreover, the NEXAFS spectra of O K-edge (Supplementary Fig. 14) for Fe–NC SAC shows no distinct features of O–Fe bonding which should appear in the region from 529 to 531 eV^61^, suggesting there is no significant O–Fe bonding in our Fe–NC SAC. All the above analyses support that Fe–N bonding is the dominated Fe state in Fe–NC SAC. The amount of Fe in Fe–NC SAC is further determined by thermogravimetric analysis (TGA) (Supplementary Fig. 15). The Fe loading of 8.9 wt% is significantly higher than those in reported SACs (Supplementary Table 2), suggesting the present strategy is able to deliver single-atomic catalysts with a high metal loading. Importantly, it should be noted that this cascade-anchoring strategy can achieve the mass production of Fe–NC SAC due to facile and manageable processing. As a demonstration, about 8 g of Fe–NC SAC is easily obtained in a one-batch synthesis (Supplementary Fig. 16) in the laboratory. XRD pattern and TEM images indicate that the product shares the similar structure to the sample characterized above (Supplementary Fig. 17).

### Insight into the formation process of Fe–NC SAC

**Fig. 4:**
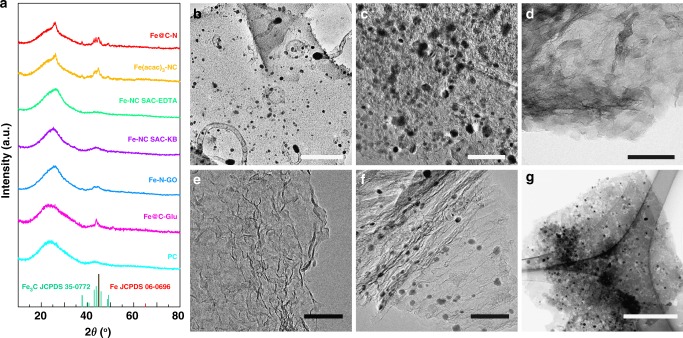
Structural characterizations of control samples. **a** XRD patterns and **b**–**g**, TEM images of Fe@C–N (**b**), Fe(acac)_3_–NC (**c**), Fe–NC SAC–EDTA (**d**), Fe–NC SAC–KB (**e**), Fe–N–GO (**f**), and Fe@C-Glu (**g**). Scale bars: 200 nm (**b**, **d**); 100 nm (**c**, **e**, **f**); 500 nm (**g**)

As mentioned above, the present cascade-anchoring strategy is able to prepare single-atomic Fe–NC materials with a high Fe loading up to 8.9 wt%. It is found that the successive protection tactics at each stage during the synthesis are essential to prevent the aggregation of Fe atoms at high loading condition. (1) Glucose chelating effect: The chelation of Fe ion with glucose is the first protection step to well isolate Fe ion in physical space. The control sample (Fe@C–N) prepared in parallel, except for no addition of glucose, shows clearly iron/iron carbide nanoparticles on carbon sheets with some nanotubes formed via Fe-catalyzed growth during the decomposition of melamine, as evidenced by XRD pattern (Fig. 4a) and TEM images (Fig. 4b and Supplementary Fig. 18). The crystallized Fe species are even observed in the sample prepared at 500 °C, without addition of glucose (XRD pattern in Supplementary Fig. 19 for control sample Fe@C–N-500). The role of chelation is corroborated by another two control experiments. If inorganic iron salt (iron (III) nitrate) is substituted with iron (III) acetylacetonate (Fe(acac)_3_), where the strong interaction between Fe ion and acetylacetone excludes the chelation of glucose and Fe ion, plenty of iron carbide nanoparticles are formed instead of isolated Fe atoms as suggested by XRD (Fig. 4a) and TEM results (Fig. 4c and Supplementary Fig. 20). Moreover, if other chelating agent such as ethylenediamine tetraacetic acid (EDTA) is used to replace glucose, XRD, TEM, and EDS-mapping results suggest that the similar Fe–NC SAC material is obtained (Fig. 4a, d and Supplementary Figs. 21 and 22). In view of the low cost, availability and solubility in water for mass production, glucose was used as chelating agents in our experiments. (2) Physical isolation of complex: The physical isolation of Fe centers is also important. The excessive amount of glucose is found to be necessary for achieving single-atomic Fe–N_x_ in the final product. The insufficient amount of glucose (such as 5:1 molar ratio of glucose: Fe) cannot keep Fe atoms away enough to prevent their aggregation during high-temperature pyrolysis so that some Fe-based nanoparticles are produced (Fe–NC–Low Glu, Supplementary Figs. 23 and 24). (3) Carbon substrate: O-rich substrate with a high surface area is critical for the synthesis of high-loading single-atomic Fe–N_x_. Commercial Ketjenblack (KB) with a comparable surface area of 1400 m^2^ g^−1^ can be used to replace PC support (Supplementary Fig. 25). XRD pattern in Fig. 4a and TEM images in Fig. 4e and Supplementary Fig. 26 imply that the similar catalyst structure is obtained. It is noted that the acid pre-treatment of KB to create O-rich surface is necessary to achieve uniform distribution of glucose–Fe complex and physically isolate Fe centers on substrate. Without acid treatment, KB is not dispersed well in aqueous solution and Fe aggregates are obtained. In contrast, if graphene oxide (GO) with a low surface area of 90 m^2^ g^−1^ is used (Supplementary Fig. 25), a number of Fe-based nanoparticles are produced as evidenced by XRD pattern (Fig. 4a) and TEM images (Fig. 4f and Supplementary Fig. 27). It is believed that such surface area is not sufficient to isolate glucose–Fe complex, leading to the aggregation of active Fe atoms during pyrolysis. (4) Cascade protection at different stages: As mentioned above, excessive glucose to chelate Fe ion and physically isolate them is required to achieve Fe–NC SAC; however, it is not sufficient. It is found that the glucose protection is only effective at a moderate temperature. The control sample prepared without addition of melamine shows no crystalline Fe species at the pyrolysis temperature up to 500 °C (Fe@C-Glu-500) (Supplementary Fig. 19), whereas increasing the pyrolysis temperature to 600 °C crystallized Fe_2_O_3_ phase can be clearly detected in XRD pattern (Fe@C-Glu-600) (Supplementary Fig. 19). Further increasing the temperature to 800 °C metallic Fe-based phase shows up in XRD pattern (Fig. 4a) of the sample (Fe@C-Glu) instead of Fe_2_O_3_ phase due to carbothermal reduction and a number of nanoparticles can be clearly identified in TEM images (Fig. 4g and Supplementary Fig. 28). Without melamine these nanoparticles can even migrate, leaving the holes in carbon substrate. It is known that melamine decomposes to N-containing species such as C_3_N_4_ etc. at over 400 °C^62^. In the synthesis of Fe–NC SAC, when the Fe–glucose complexes start to decompose and release Fe atoms at elevated temperature, the surrounding abundant active CN_x_ species decomposed from excessive amount of melamine will instantly capture these active Fe atoms by forming Fe–N bonding as Fe–N_x_ species since it is an energy-favorable process, similar to the case of the formation of Pd–N^63^. These Fe–N_x_ species will be subsequently incorporated into the carbon network newly evolved from the graphitization of glucose and melamine during pyrolysis, preventing them from aggregation. The single-atomically dispersed Fe–N_x_ sites have been evidenced by the above-mentioned analyses.

### Electrocatalytic performance evaluation of Fe–NC SAC

**Fig. 5:**
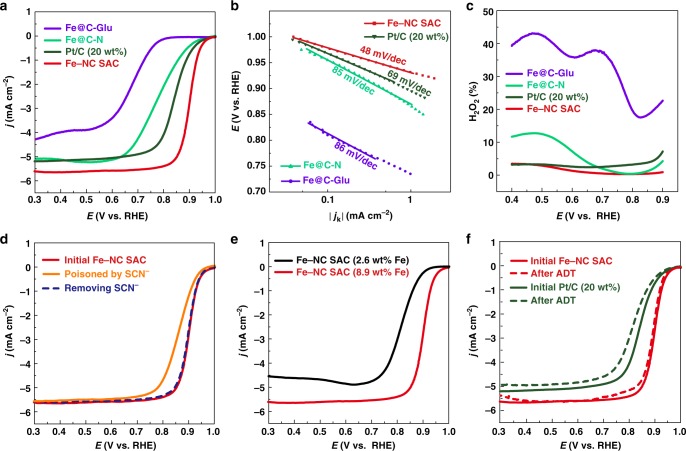
Evaluation of electrocatalytic performance of Fe–NC SAC for ORR. **a** Steady-state ORR polarization curves of Fe–NC SAC, Pt/C and control samples (Fe@C–N and Fe@C-Glu). **b** Corresponding Tafel plots. **c** Hydrogen peroxide yield. **d** Steady-state ORR polarization curves of Fe–NC SAC recorded in O_2_-saturated 0.1 M KOH with or without poisoning by 0.01 M SCN^−^, and one collected after removing SCN^−^. **e** Steady-state ORR polarization curves of Fe–NC SAC with different Fe loading. **f** Steady-state ORR polarization curves of Fe–NC SAC and Pt/C before and after 5000 potential scanning cycles in O_2_-saturated 0.1 M KOH

As a demonstration of the potential applications of M–NC SACs, the electrocatalytic activity of Fe–NC SAC for ORR was evaluated and compared with benchmark Pt/C catalyst and control samples (Fe@C–N, Fe@C-Glu, and C–N-Glu). As shown in Fig. 5a and Supplementary Table 3, the state-of-the-art commercial Pt/C (20 wt%) exhibits a good ORR activity in terms of an onset potential (defined by the potential at which the current density reaches 0.1 mA cm^−2^)^64,65^ of 0.96 V and a half-wave potential of 0.85 V. While the present Fe–NC SAC obviously shows a positively shifted onset potential (0.98 V) and a half-wave potential (0.90 V), which are 20 and 50 mV more positive than those for Pt/C catalyst and superior to most of non-precious metal ORR electrocatalysts (Supplementary Table 4). The mass activity of Fe–NC–SAC is calculated to be 9.0 A g^−1^ at 0.90 V. What is more, the Fe–NC SAC shows a high kinetic mass current of 100.7 A g^−1^ at 0.90 V, 65 A g^−1^ larger than Pt/C (35.7 A g^−1^). In contrast, control sample Fe@C–N (prepared without glucose), Fe@C-Glu (prepared without melamine), and C–N–Glu (prepared without iron) demonstrate poor ORR electrocatalytic activities (Supplementary Fig. 29). The superior ORR electrocatalytic activity of Fe–NC SAC is further confirmed by the smallest Tafel slope (48 vs. 69 mV dec^−1^ for Pt/C) (Fig. 5b) and the lowest H_2_O_2_ yield (below 3.5% at the whole potential range) (Fig. 5c). These results indicate that ORR process on Fe–NC SAC follows a four-electron pathway with a high electrocatalytic efficiency for ORR.

To get insight into the active sites for ORR in Fe–NC SAC, additional control experiments were carried out. Although there is still debate about the most active sites for ORR in Fe–N–C catalysts due to complicate catalyst structures, Fe–N_x_ site is generally considered to play an important role in catalyzing ORR. It has been reported that SCN^−^ ion is able to strongly interact with Fe center, thus poisoning Fe–N_x_ coordination site^66^. Since the coordination of Fe–SCN is stable in the acidic condition but not in the alkaline medium, we first test the ORR activity of Fe–NC SAC in O_2_-saturated 0.1 M HClO_4_ containing 0.01 M SCN^−^. The result shows that the half-wave potential decrease distinctly by about 116 mV comparing with the curve measured without addition of 0.01 M SCN^−^ (Supplementary Fig. 30), which can be ascribed to the blocking of Fe–N_x_ active sites by SCN^−^. Moreover, when rinsing this pre-poisoned electrode to pH = 7 and re-testing it in O_2_-saturated 0.1 M KOH, the half-wave potential negatively shifts by about 30 mV compared with the values measured in 0.1 M KOH without SCN^−^ (Fig. 5d)^67,68^. It is noted that polarization curve can gradually recover to its original state during the measurements since the blocked Fe–N_x_ coordination sites can be gradually released as the dissociation of Fe–SCN^−^ in KOH (Fig. 5d). These results suggest that Fe–N_x_ sites should be the most active sites in Fe–NC SAC in view that only two possible active sites exist, i.e. Fe–N_x_ sites and N-doped carbon. To further demonstrate the role of Fe–N_x_ sites, we prepared another control sample Fe–NC SAC with a low Fe loading via reducing the amount of Fe source and keeping all other conditions same. The characterizations indicate that the similar catalyst to Fe–NC SAC was achieved except for the low Fe loading of 2.6 wt% (*vs*. 8.9 wt% for Fe–NC SAC) (Supplementary Fig. 31). The electrochemical measurements show that the ORR electrocatalytic activity is significantly attenuated in terms of 80 mV negatively shifted half-wave potential and decreased limiting current density (Fig. 5e). This result corroborates that Fe–N_x_ sites in Fe–NC SAC are the efficient active sites for delivering a high ORR activity and the loading of Fe–N_x_ sites affects the activity. Furthermore, it is noted that Fe–NC SAC also shows a better ORR electrocatalytic activity than the reference sample FePc/C which was prepared by loading FePc on carbon substrate with a similar Fe loading (see more details in Supplementary Fig. 32). This could be ascribed to the severe aggregation of FePc and the possible differences in the environments of Fe–N_x_ centers for our Fe–NC SAC and reference FePc/C.

Durability is another important criterion for assessing electrocatalyst performance. The durability of Fe–NC SAC was evaluated using accelerated durability test (ADT) by cyclic voltammetry experiment from 0.6 to 1.0 V at 50 mV s^−1^ in O_2_-saturated 0.1 M KOH. As shown in Fig. 5f, polarization curves recorded after 5000 cycles for Fe–NC SAC display a negligible degradation for half-wave potential and limiting current density, indicating its superior durability in alkaline medium. TEM and EDS-mapping images reveal that no Fe aggregation is observed, and the atomic dispersion of Fe–N_x_ sites retains in Fe–NC SAC after ADT (Supplementary Fig. 33). By contrast, the commercial Pt/C catalyst shows a 20 mV loss in half-wave potential and appreciable reduction of limiting current density after ADT. The significant activity degradation for Pt/C catalyst should be attributed to the serious dissolution/agglomeration of Pt nanoparticles during ADT, as shown in Supplementary Figs. 34 and 35^69^.

### Demonstration for general synthesis of M–NC SACs

**Fig. 6:**
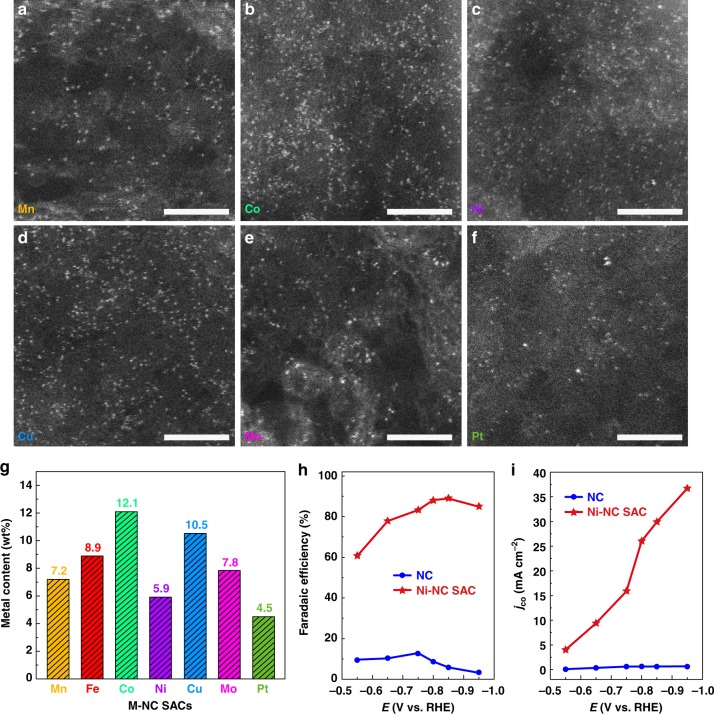
Atomic structure characterizations and loading analysis of M–NC SACs. **a**–**f** HAADF–STEM images for Mn–NC SAC (**a**), Co–NC SAC (**b**), Ni–NC SAC (**c**), Cu–NC SAC (**d**), Mo–NC SAC (**e**), and Pt–NC SAC (**f**). Scale bars, 3 nm (**a**–**f**). **g** Metal loading in M–NC SACs. **h** Faradaic efficiency of CO, and **i**, *j*_co_ for Ni–NC SAC and control sample (NC)

Inspired by the simplicity and general applicability of the present cascade-protection strategy, we easily extend it to prepare a wide range of other M–NC SACs (M = Mn, Co, Ni, Cu, Mo, Pt, etc.) with a high metal loading. As shown in Fig. 6a–f and Supplementary Fig. 36, the white spots in a size of single atom in HAADF–STEM images indicate that Mn, Co, Ni, Cu, Mo, and Pt are single-atomically dispersed in the PC substrate. A couple of spots with different size could be ascribed to the metal atom imaged at different focusing planes or possible atom cluster. XRD patterns prove that no crystallized metal-based phases are detected (Supplementary Fig. 37). EDS spectra, corresponding mapping images and EELS-mapping images confirm the existence of element metal and nitrogen as well as their homogenous distribution (Supplementary Figs. 38–45). These results suggest that these M–NC SACs should share the similar structure to Fe–NC SAC. TGA analyses give the metal loading from 12.1 to 4.5 wt% (Fig. 6g and Supplementary Fig. 46). The variation could be ascribed to the differences in the interaction of glucose and metal ions as well as the carbon loss in the presence of different metals. The exploration for the applications of these M–NC SACs are expected in view of the high density of single-atomically dispersed metal–N_x_ active centers. For example, Ni–NC SAC exhibits the potential for CO_2_ reduction to CO and shows an 89% of Faraday efficiency at −0.85 V with a 30 mA cm^−2^ of current density for CO (Supplementary Fig. 47 and Fig. 6h, i). This high current density outperforms most reported Ni-based single-atom catalysts, which should be attributed to the high-density Ni-based active sites with a Ni loading of 5.9 wt% in Ni–NC SAC (Supplementary Tables 5 and 6).

## Discussion

In summary, a cascade-protection strategy is developed to synthesize single-atomic metal–N_x_ sites on N-doped carbon with a high metal loading up to 12.1 wt%. The single-atomic dispersion of metal atoms and the formation of metal–N_x_ sites are evidenced by the different analytic techniques, including HAADF–STEM, EELS, XANES, EXAFS, and NEXAFS etc. The formation process of single-atomic metal–N_x_ sites and the effectiveness of the cascade-protection strategy are systematically investigated by a series of control experiments. The results suggest that the chelation of glucose and Fe ion, physical isolation of glucose–Fe complex via excessive glucose, support with sufficient O-species and surface area to anchor and isolate glucose–Fe complex, and the binding with N-species at elevated pyrolysis temperature are required to achieve the high-loading single-atomic Fe–N_x_ sites. As a demonstration, the electrocatalytic performance of Fe–NC SAC for ORR is evaluated. Benefiting from the high-loading Fe–N_x_ active sites and their single-atomic dispersion for exposing each site, Fe–NC SAC exhibits a superior ORR electrocatalytic activity and durability with a half-wave potential of 0.90 V and a kinetic mass current of 100.7 A g^−1^ at 0.90 V, 50 mV and 65 A g^−1^ higher than state-of-the-art Pt/C, respectively. The poisoning and loading-dependent experiments indicate that such ORR performance should be from Fe–N_x_ sites. The mass production and scalability of the present strategy is demonstrated by synthesis over 8 g of such Fe–NC SAC with consistent ORR activity in a single-lab batch. The universal syntheses of other M–NC SACs (M = Mn, Co, Ni, Cu, Mo, Pt, etc.) demonstrate that such strategy can be easily adapted to apply different chelating agents, substrates, and metal sources for a wide range of metal–NC SACs. In view of high-loading metal–N_x_ coordination sites in single-atomic level, these materials are expected for diverse applications, including electrocatalysis and heterogeneous catalysis etc.

## Methods

### Synthesis of PC support

In total, 8 mmol of potassium citrate (Alfa Aesar Co., Ltd.) was pyrolyzed at 800 °C for 1 h in a tube furnace and Ar atmosphere. The black solid product was washed with H_2_SO_4_ solution (0.5 M) (Alfa Aesar Co., Ltd.) and water (18.2 MΩ) to remove inorganic impurities. After drying at 60 °C, the PC support was achieved.

### Synthesis of Fe–NC SAC

In total, 60 mg of PC, 0.3 mmol of iron (III) nitrate nonahydrate (Alfa Aesar Co., Ltd.), and 6.7 mmol of α-D-glucose (Sinopharm Chemical Reagent Co., Ltd.) were dispersed in 5 mL of ultrapure water, and sonicated for 30 min to get a homogenous black suspension. The slurries were harvested after washing with water and drying at 60 °C, and then grounded together with melamine (Alfa Aesar Co., Ltd.) at a mass ratio of 1:5. The obtained powder was placed into a tube furnace and heated to 800 °C under Ar flow (100 sccm). After 2 h of pyrolysis, the black Fe–NC SAC was obtained. M–NC SACs (M = Mn, Co, Ni, Cu, Mo, and Pt) were prepared via the same procedures, except for using manganese nitrate hexahydrate (Alfa Aesar Co., Ltd.), cobalt nitrate hexahydrate (Alfa Aesar Co., Ltd.), nickel nitrate hexahydrate (Alfa Aesar Co., Ltd.), cupric nitrate hemipentahydrate (Alfa Aesar Co., Ltd.), ammonium molybdate tetrahydrate (Alfa Aesar Co., Ltd.), and (hydro)chloroplatinic acid (Alfa Aesar Co., Ltd.) as the metal precursor, respectively.

### Synthesis of control samples

(1) Fe–NC SAC–EDTA was synthesized in parallel by the same method as that for Fe–NC SAC, except for using the same mass amount of EDTA (Alfa Aesar Co., Ltd.) instead of glucose. Fe(acac)_3_–NC was prepared in parallel by the same method as that for Fe–NC SAC, except for using the same molar amount of Fe(acac)_3_ (Alfa Aesar Co., Ltd.) instead of iron (III) nitrate nonahydrate. (2) Fe–NC SAC–KB and Fe–N–GO were prepared in parallel by the same method as that Fe–NC SAC except for using the same mass amount of commercial KB (1400 m^2^ g^−1^) (Ketjenblack EC-600JD, Akzo Nobel, Inc.) and GO (90 m^2^ g^−1^) (Nanjing XFNANO Materials Tech. Co.) instead of PC support, respectively. (3) Fe@C-Glu was obtained in parallel by the same synthesis route as that for Fe–NC SAC, except for no addition of melamine; the samples pyrolyzed at 500, 600, and 800 °C are designated as Fe@C-Glu-500, Fe@C-Glu-600, and Fe@C-Glu, respectively. (4) Fe@C–N was prepared in parallel by the same synthesis route as that for Fe–NC SAC, except for no addition of glucose; the samples pyrolyzed at 500 and 800 °C are designated as Fe@C–N-500 and Fe@C–N, respectively. (5) Fe–NC–Low Glu was prepared in parallel by the same method as that Fe–NC SAC except for using 1.5 mmol glucose instead of 6.7 mmol glucose. (6) C–N–Glu was prepared in parallel by the same synthesis route as that for Fe–NC SAC, except for no addition of iron source. (7) FePc/C was prepared by dispersion 60 mg of PC and 42 mg of FePc (Alfa Aesar Co., Ltd.) in 5 mL of dimethyl formamide (Alfa Aesar Co., Ltd.), followed by sonication for 30 min to get a homogenous black suspension. The product was collected after washing and drying. (8) NC was synthesized in parallel by the same method as that Fe–NC SAC, except for no addition of iron source and glucose.

### A scale-up synthesis of Fe–NC SAC

The product was prepared in parallel by the same synthesis route as that for Fe–NC SAC, except for increasing the amount of carbon substrate, iron (III) nitrate nonahydrate, glucose, and ultrapure water to 5 g, 24.8 mmol, 0.56 M and 420 mL, respectively.

### Catalyst characterizations

XRD patterns were recorded on Regaku D/Max-2500 (Rigaku Co., Japan) diffractometer equipped with a Cu Kα1 radiation (*λ* = 1.54056 Å). The morphologies were characterized by SEM on S4800 (JEOL, Japan) and TEM on JEM-2100F (JEOL, Japan) equipped with an EDS detector. HAADF–STEM and EELS mapping observations were carried out on a JEOL ARM200F (JEOL, Japan) STEM operated at 200 kV with cold-filled emission gun and double hexapole Cs correctors (CEOS GmbH, Germany). The attainable spatial resolution defined by the probe-forming objective lens is better than 80 picometers. Nitrogen adsorption–desorption isotherms were collected on a Quadrasorb SI-MP system (Quantachrome, USA) at 77 K. The specific surface area was calculated by Brunauer Emmett Teller method. The pore size distribution and pore volume were calculated using DFT method. XPS spectra were recorded on an ESCALab220i-XL electron spectrometer (VG Scientific, UK) using an Al Kα radiation. Raman spectra were obtained on Lab-RAM HR Evolution (Horiba Scientific, Japan) with a laser excitation wavelength of 532 nm. FTIR experiments were performed using Thermo Fisher Nicolet iN10 FTIR microscope (Thermo Nicolet Corp., USA). The metal loading was measured via TGA on a Netzsch DSC214 instrument (NETZSCH, Germany) from 40 to 800 °C under air flow with a ramp of 10 °C min^−1^. XANES and EXAFS of the Fe K-edges were acquired on the XAFS station of the 14W1 beam line of the Shanghai Synchrotron Radiation Facility. The fluorescence mode was used to record the X-ray absorption spectra of Fe K-edges. Data were recorded by using a Si (111) double-crystal monochromator. The back-subtracted EXAFS function was converted into *k* space and weighted by *k*^3^ to compensate for the diminishing amplitude due to the decay of the photoelectron wave. The Fourier transforming of the k^3^-weighted (for Fe) EXAFS data was performed in the range of k = 3–12 Å^−1^ using a Hanning function window to get the radial distribution function. The NEXAFS spectra were collected at Beamline 8.0 of Advanced Light Source in Lawrence Berkeley National Lab via a total fluorescence yield mode with a probing depth of ~100 nm. The samples were thoroughly outgassed before measurements.

### Electrochemical measurements. ORR Tests

All ORR electrochemical measurements were recorded on a rotating ring-disk electrode rotator (RRDE-3A) (ALS, Japan) by a standard three-electrode cell system connected to an electrochemical workstation (Autolab PGSTAT 302N) (Metrohm, Netherlands) at room temperature. A rotating ring-disk electrode (RRDE) (4 mm in diameter) with catalytic material acted as the working electrode; and an Ag/AgCl electrode (saturated KCl solution) and a graphite rod were employed for the reference and counter electrode, respectively. The working electrodes were prepared as follows: all non-precious homogeneous ink was formed by mixing 2 mg of catalysts and 800 µL of ethanol (Beijing Chemical Reagent Factory) in a glass vial and sonicated for 30 min. The 30 µL of ink and 2 µL 0.5 wt% of Nafion solution (Alfa Aesar Co., Ltd.) were dropped on polished RRDE to get catalysts loading of 600 µg cm^−2^ and dried 10 min in the air. For comparison, commercial Johnson–Matthey Pt/C (20 wt%) was also measured with loading of 25.5 µg_Pt_cm^−^^2^. The accelerated durability tests of Fe–NC SAC and commercial Pt/C were performed in the O_2_-saturated 0.1 M KOH (Alfa Aesar Co., Ltd.) solution by cycling the catalysts between 0.6 and 1.0 V at 50 mV s^−1^, referring to the protocol from USA Department of Energy. Poisoning experiment for Fe–NC SAC was first executed in O_2_-saturated 0.1 M HClO_4_ (Alfa Aesar Co., Ltd.) with addition of 0.01 M NaSCN (Alfa Aesar Co., Ltd.). The remarkably depression of catalytic activity can be seen. After that, the working electrode with poisoned Fe–NC SAC was rinsed thoroughly and measured again in 0.1 M KOH under O_2_ atmosphere. All ORR measurements were collected at a scan rate of 10 mV s^−1^ at a rotation speed of 1600 rpm. Before each ORR test, the electrolyte was purged with oxygen at least for 30 min. All non-Pt catalysts were scanned in the N_2_-saturated electrolyte. The obtained background voltammograms were subtracted from that measured in the O_2_-saturated electrolyte before each ORR measurement.

The yield of H_2_O_2_ on different catalysts was calculated by the following equation:

1 $${\mathrm{H}}_2{\mathrm{O}}_2\% = {\mathrm{200}} \ast \frac{I_{\mathrm{R}}/N}{I_{\mathrm{R}}/N + I_{\mathrm{D}}}$$

where *I*_R_ and *I*_D_ are the ring and disk currents, respectively, and *N* is the ring collection efficiency. The *N* value was measured to be 42.4%.

### CO_2_ reduction tests

All electrochemical measurements for CO_2_ reduction were carried out in a gas-tight cell with two-compartments separated with a Nafion membrane (Nafion®115, DuPont, Inc.). A same standard three-electrode cell system as that for ORR was used to collect electrochemical data. In a typical prepared process of the working electrode, 600 µL of the mixing ink, which was obtained by dispersing 1 mg catalyst in 30 µL 0.5 wt% of Nafion solution and 570 µL of ethanol solution, was loaded on two sides of a carbon cloth (Alfa Aesar Co., Ltd.) with 1 × 1 cm^2^. During the CO_2_ reduction experiments, all measurements were collected at a scan rate of 10 mV s^−1^ in N_2_-saturated 0.5 M KHCO_3_ (Alfa Aesar Co., Ltd.) or CO_2_-saturated 0.5 M KHCO_3_ electrolyte. The gas phase composition was analyzed by gas chromatograph (Agilent 7890B, USA) every 1 h. The separated gas products were measured by a thermal conductivity detector (for H_2_) and a flame ionization detector (for CO).

## Supplementary information

Supplementary Information

Peer Review

## Data Availability

The data that support the findings of this study are available from the corresponding author upon reasonable request.
