# Supplementary material for: Cascade anchoring strategy for general mass production of high-loading single-atomic metal-nitrogen catalysts
Source: Nat Commun. 2019 Mar 20;10:1278. doi: 10.1038/s41467-019-09290-y (PMC6426845; doi:10.1038/s41467-019-09290-y)
Supplement: Supplementary file 1 — Supplementary Information [file 41467_2019_9290_MOESM1_ESM.pdf]

Supplementary Information

Cascade Anchoring Strategy for General Mass Production of High-Loading Single-Atomic Metal-Nitrogen Catalysts

Lu Zhao[1,2,†], Yun Zhang[1,3,†], Lin-Bo Huang[1,2], Xiao-Zhi Liu[2,4], Qing-Hua Zhang[4], Chao He[1,2], Ze-Yuan Wu[1,2], Lin-Juan Zhang[5], Jinpeng Wu[6], Wanli Yang[6], Lin Gu[4], Jin-Song Hu[1,2,*] and Li-Jun Wan[1,2]

Contents:

Supplementary Figures 1 to 47

Supplementary Tables 1 to 6

[1] Beijing National Laboratory for Molecular Sciences (BNLMS), CAS Key Laboratory of Molecular Nanostructure and Nanotechnology, Institute of Chemistry, Chinese Academy of Sciences, Beijing 100190, China. [2] University of Chinese Academy of Sciences, Beijing 100049, China. [3] College of Chemistry and Materials Science, Sichuan Normal University, Chengdu 610068, China. [4] Beijing National Research Center for Condensed Matter Physics, Collaborative Innovation Center of Quantum Matter, Institute of Physics, Chinese Academy of Sciences, Beijing 100190, China. [5] Shanghai Synchrotron Radiation Facility, Shanghai Institute of Applied Physics, Chinese Academy of Sciences, Shanghai 201800, China. [6] Advanced Light Source, Lawrence Berkeley National Laboratory, Berkeley California 94720, United States [†] These authors contributed equally to this work. * e-mail: hujs@iccas.ac.cn.

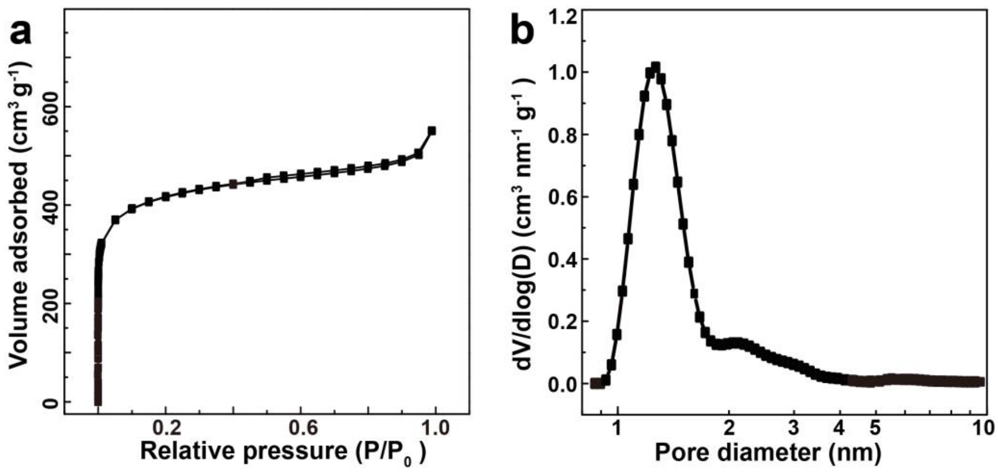

Supplementary Figure 1. a, N_2 adsorption-desorption isotherm, and **b**, Corresponding pore size distribution curve for PC support.

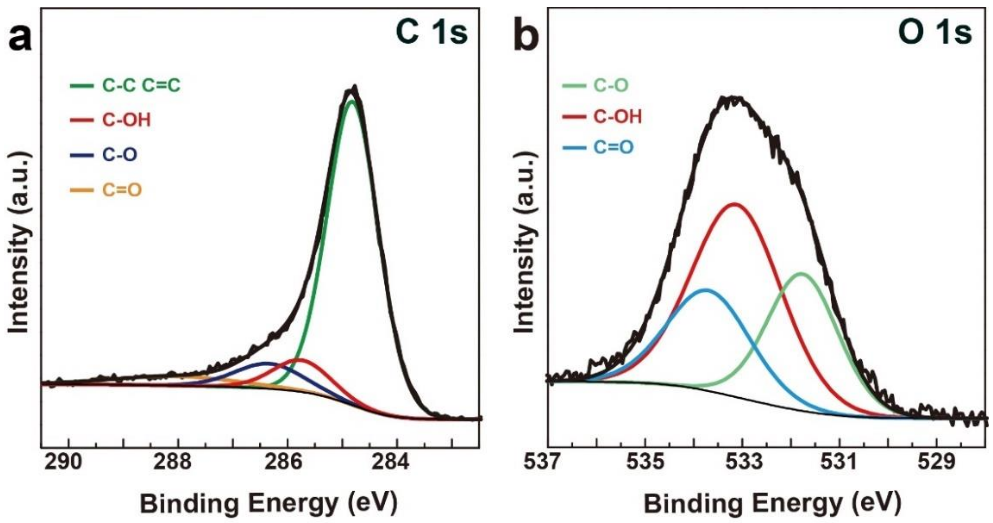

Supplementary Figure 2. a, b XPS spectra of the as-prepared PC: C 1s (**a**) and O 1s (**b**).

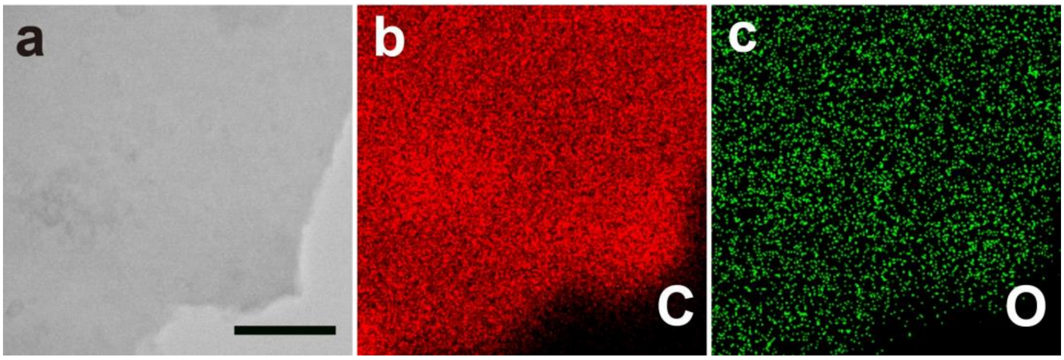

Supplementary Figure 3. a, STEM image and **b**, **c**, EDS mapping images of PC: C (**b**) and O (**c**). Scale bar, 200 nm (**a**).

Density Functional Theory (DFT) calculations on the chelation of α-D-glucose and iron (III)

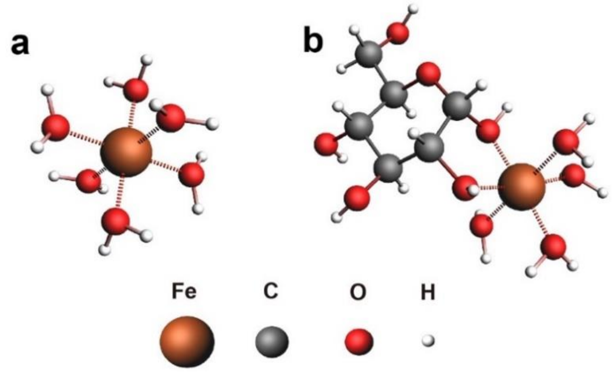

Supplementary Figure 4. a, b, Illustration of hydrated iron (III) ion (**a**) and hydrated α-D-glucose-iron (III) complex (**b**).

The chelation of D-glucose and Fe(III) ions to form D-glucose-Fe(III) complex has been reported in the literature[1]. To investigate the chelation between α-D-glucose and iron (III) in our experiments, DFT calculations were further performed. Generally, Fe(III) ion exists in form of hydrated Fe(III) in aqueous solution, where Fe(III) is usually chelated with six H_2O (Supplementary **Fig.** 4a)[2]. If α-D-glucose presents in the solution, hydroxyl groups of α-D-glucose will chelate with hydrated iron ion. For demonstrating this reaction in DFT calculations, two hydroxyl groups next to ether group in one α-D-glucose molecule were modeled to replace two H_2O molecules in hydrated iron ions according to the reported structure of metal-glucose adduct (Supplementary **Fig.** 4b)[1, 3]. This reaction can be expressed as follows:

$$Fe^{3+}(H_2O)_6 + \alpha\text{-D-glucose} \rightarrow Fe^{3+}(\alpha\text{-D-glucose})(H_2O)_4 + H_2O \quad (1)$$

According to DFT calculations, the free energy of this reaction is ΔG = - 6.12 Kcal mol^{-1}. This result demonstrates that the above reaction can spontaneously occur to form hydrated α-glucose-iron (III) complex, corroborating the chelating interaction between Fe(III) and glucose.

Calculation Methods. DFT calculations were carried out in this work by the Amsterdam Density Functional program package. The exchange and correlation energy are described with GGA-PBE. A triple-ζ plus polarization basis set is employed for all elements. The criterion of self-consistent convergence of total energy is set as 0.001 eV/atom. To save calculation time, the frozen core technology is implemented. Since iron are transition metals, the relativistic effect is also considered. The change of the free energy could be calculated as follows:

$$\Delta G = \Delta E + \Delta \mathrm{ZPE} - T\Delta S - neU + kTln10 \times \Delta \mathrm{pH} \quad (2)$$

where ΔE is the reaction energy, ZPE is the zero-point energy, T is temperature, and S is entropy. The forth term is the effect of a bias involving electrons in the electrode, n is the electron number, and U is the electrode potential relative to the standard hydrogen potential. The fifth term is the change of free energy contributed from the pH change (H^+ concentration).

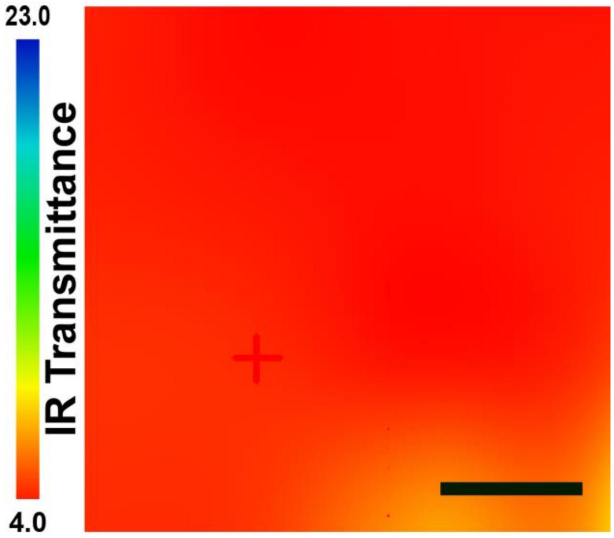

Supplementary Figure 5. Fourier-transform infrared spectroscopy (FTIR) transmittance image at 3382 cm^{-1} of PC after loading glucose and glucose-chelated Fe complex. The absorption at 3382 cm^{-1} can be assigned to the OH stretching vibrations of glucose. Scale bar, 40 µm.

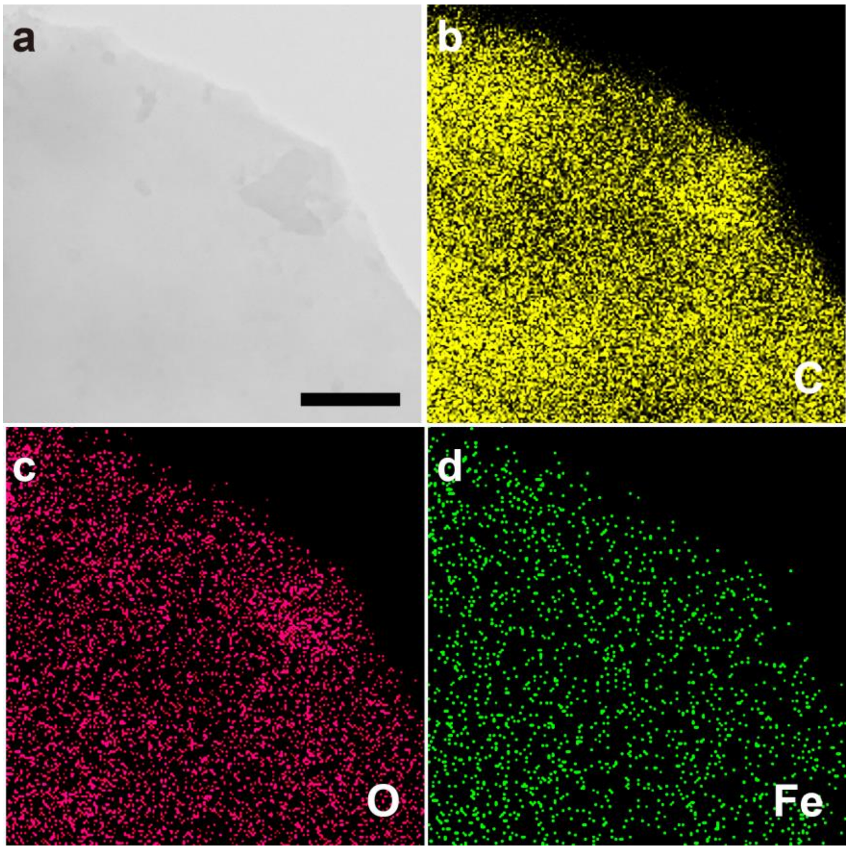

Supplementary Figure 6. a, STEM image and **b-d**, EDS mapping images of PC after loading glucose and glucose-chelated Fe complex: C (**b**), O (**c**), and Fe (**d**). Scale bar, 250 nm (**a**).

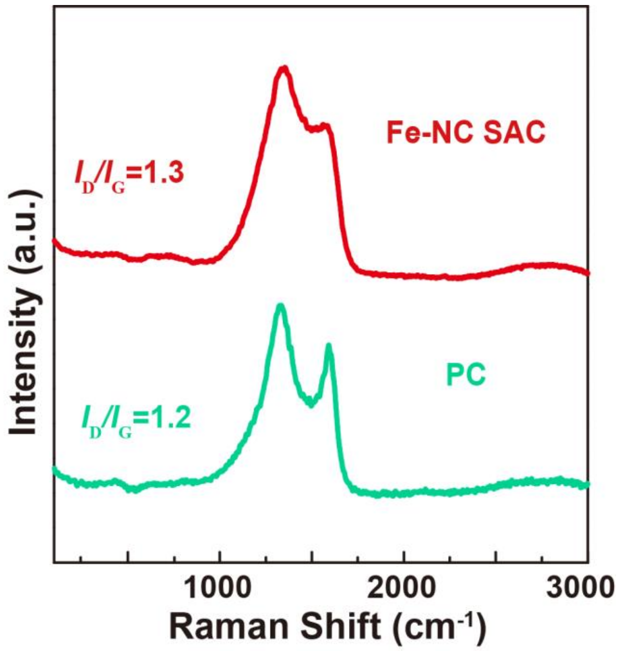

Supplementary Figure 7. Raman spectra of Fe-NC SAC and PC support (PC went through the same pyrolysis).

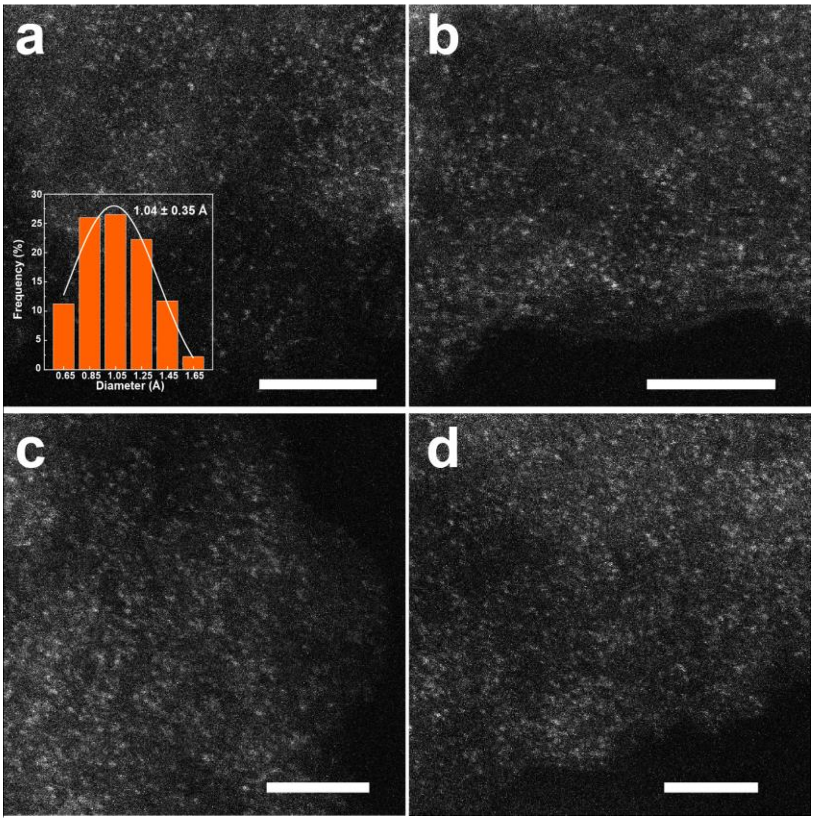

Supplementary Figure 8. a-d, Supplementary HAADF-STEM images of Fe-NC SAC. Inset in **a** is the size distribution of bright spots in HAADF-STEM images. Scale bars, 3 nm (**a-d**).

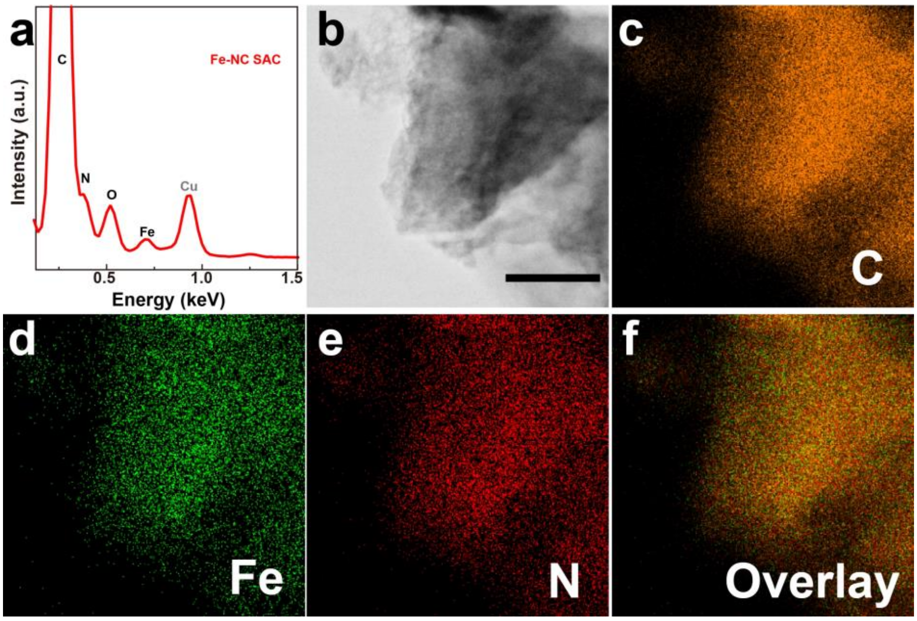

Supplementary Figure 9. a, EDS spectrum, **b**, STEM image, and **c-f**, EDS mapping images of Fe-NC SAC: C (**c**), Fe (**d**), N (**e**), and integrated elemental mapping image (**f**) of Fe-NC SAC. The signal of Cu in the spectrum comes from TEM Cu grid. Scale bar, 200 nm (**b**).

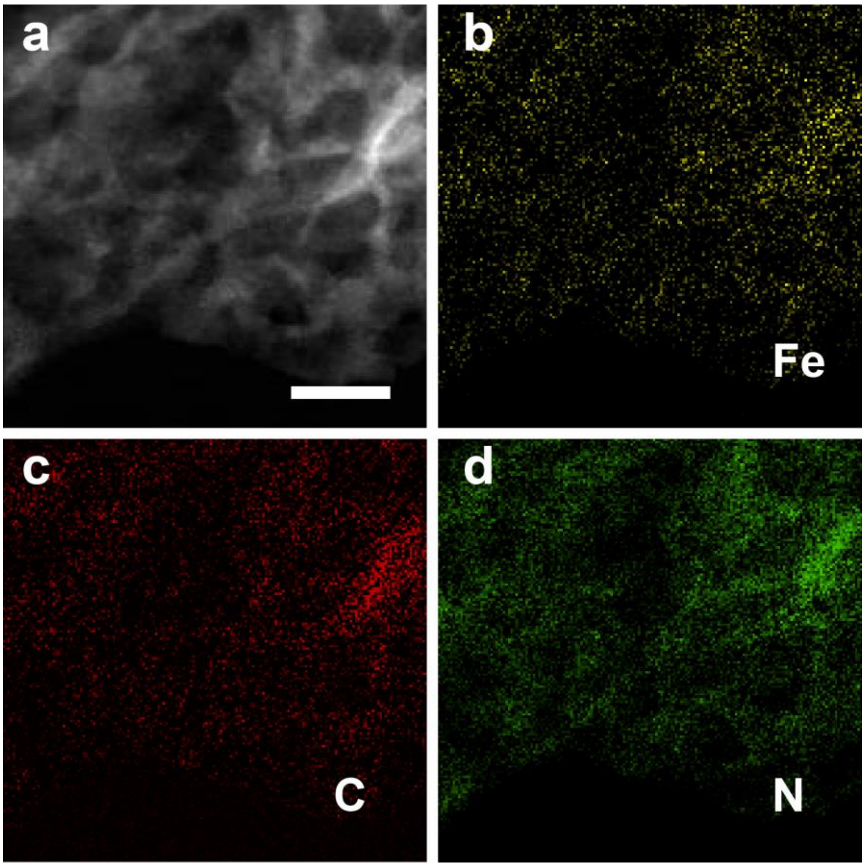

Supplementary Figure 10. a, HAADF-STEM image, and **b-d**, EELS mapping images of Fe (**b**), C (**c**), and N (**d**) for Fe-NC SAC. Scale bar, 20 nm (**a**).

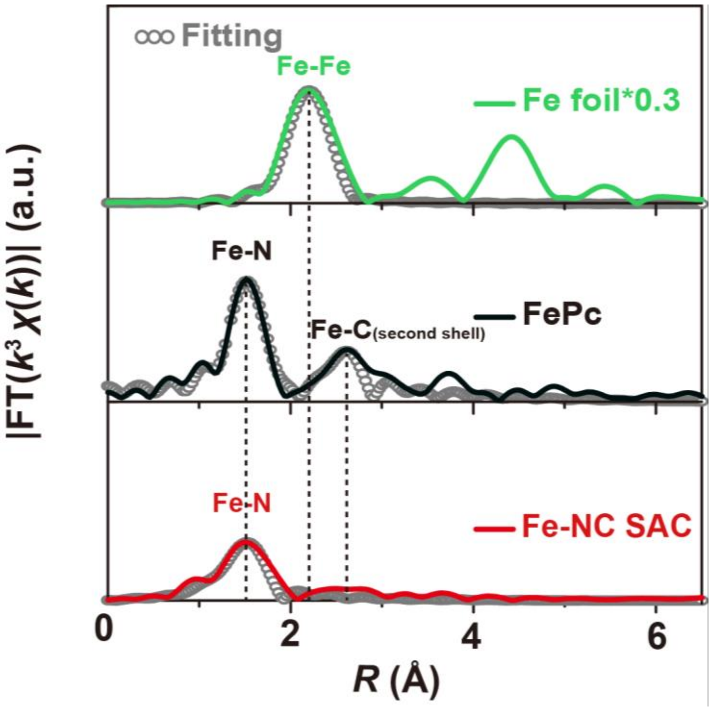

Supplementary Figure 11. Fourier transform of Fe K-edge EXAFS spectra in *R* space and the corresponding fittings for Fe-NC SAC and reference samples (FePc and Fe foil).

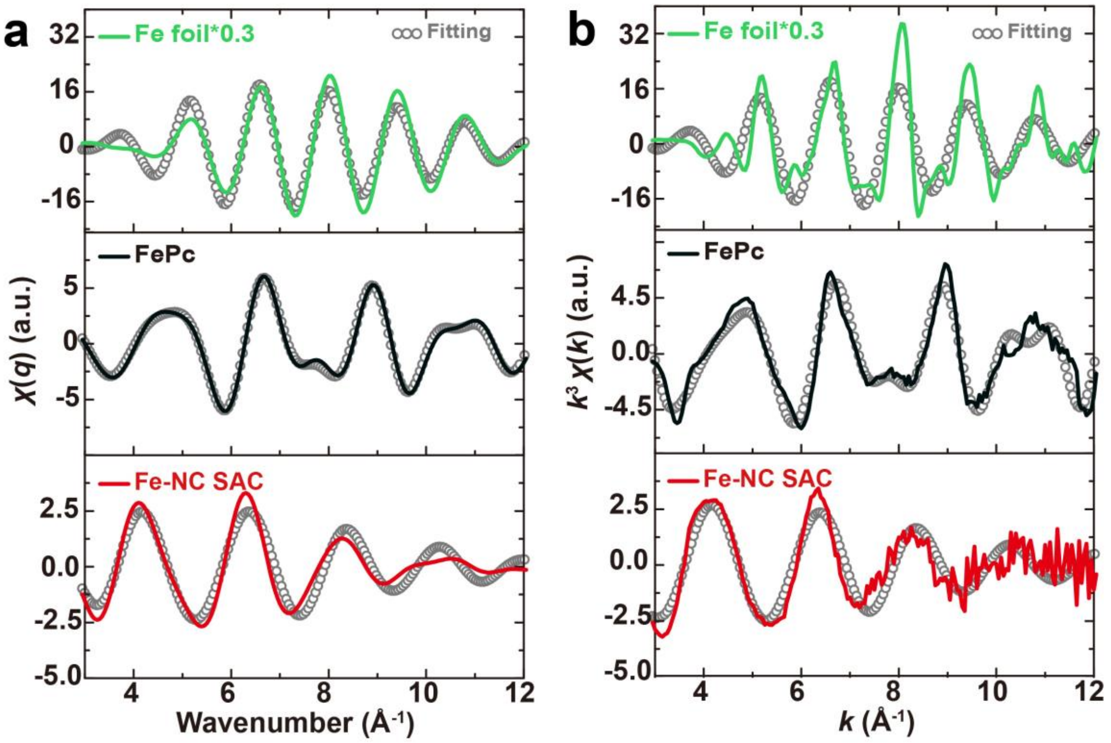

Supplementary Figure 12. **a**, **b**, Fourier transform of Fe K-edge EXAFS spectra in *q* (**a**) and *k* (**b**) space and the corresponding fittings for Fe-NC SAC and reference samples (FePc and Fe foil).

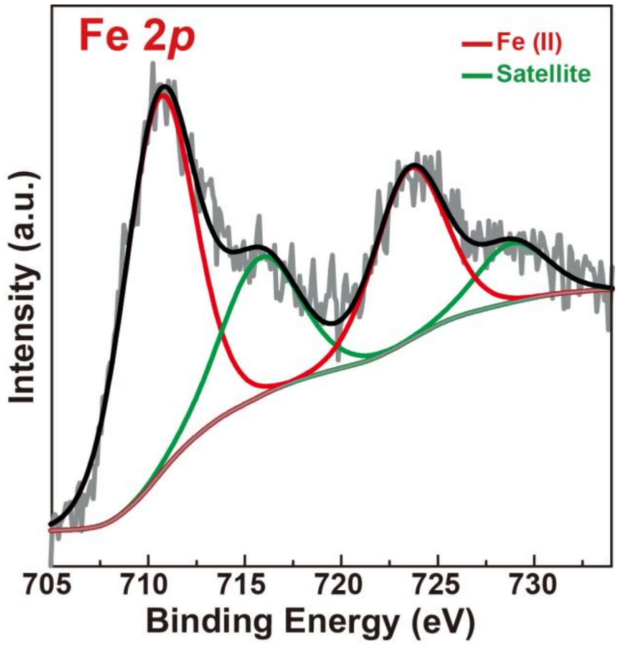

Supplementary Figure 13. Deconvoluted Fe 2*p* XPS spectrum of Fe-NC SAC.

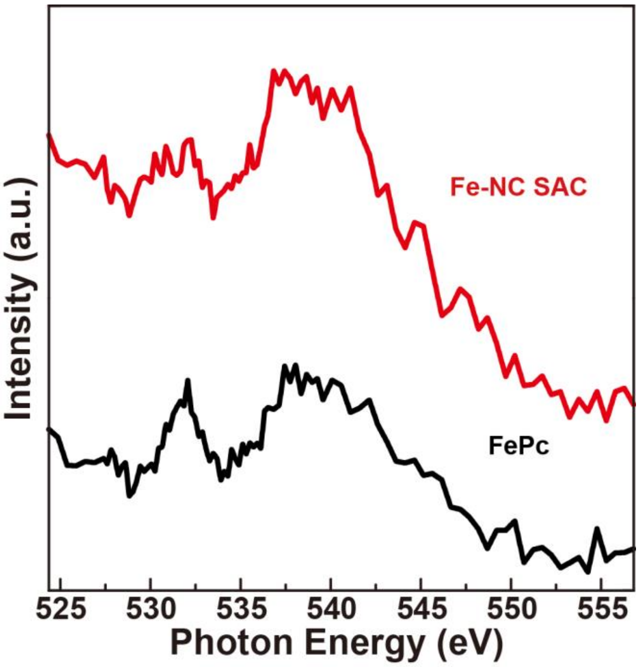

Supplementary Figure 14. O K-edge NEXAFS spectra of Fe-NC SAC and reference FePc.

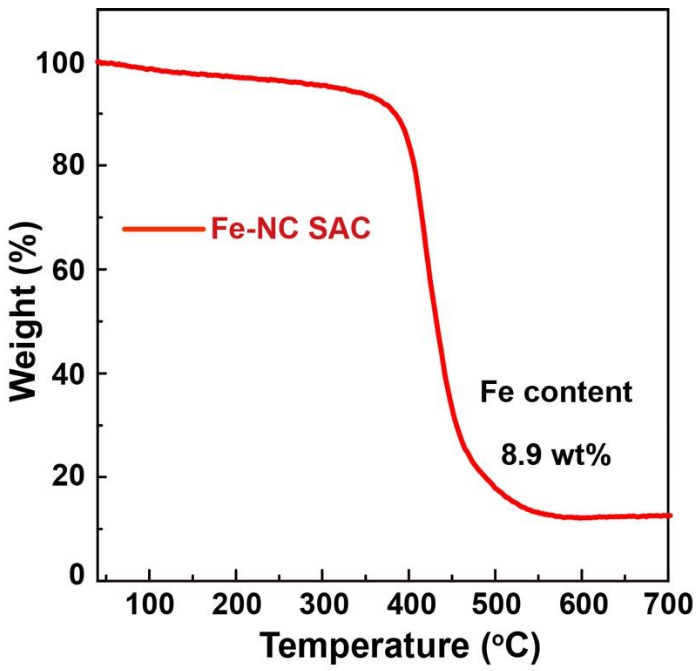

Supplementary Figure 15. TGA curve collected in air atmosphere for Fe-NC SAC.

As shown in this TGA curve, the initial weight loss below 100 °C should be attributed to the evaporation of adsorbed H_2O. The weight loss between 300 and 550 °C comes from the carbon combustion. The product after heating to 700 °C is Fe_2O_3 and its corresponding weight is 12.71 wt%. Accordingly, the Fe content is calculated basis on the following equation:

m % (Fe) = residual mass * M (Fe) / M (Fe_2O_3)

=12.71 wt% * (55.847*2) / (55.847*2+16.000*3)
=8.9 wt% (3)

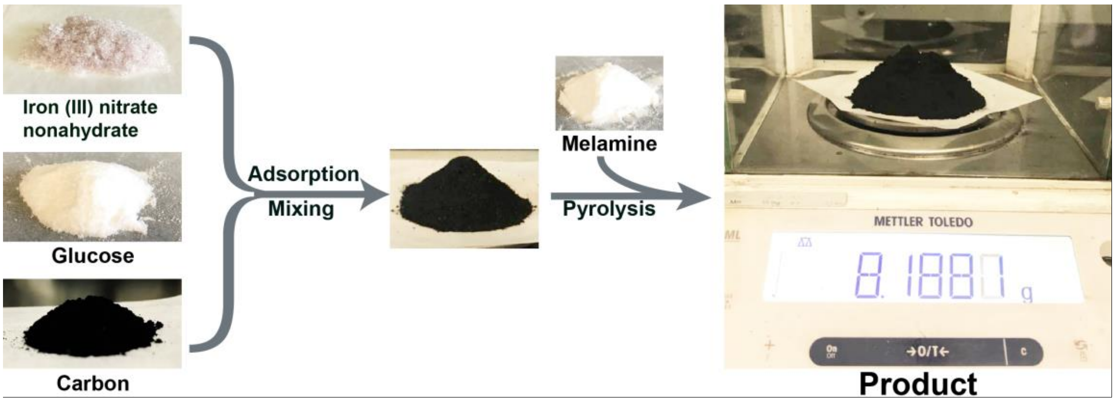

Supplementary Figure 16. Scheme for a scale-up synthesis of Fe-NC SAC.

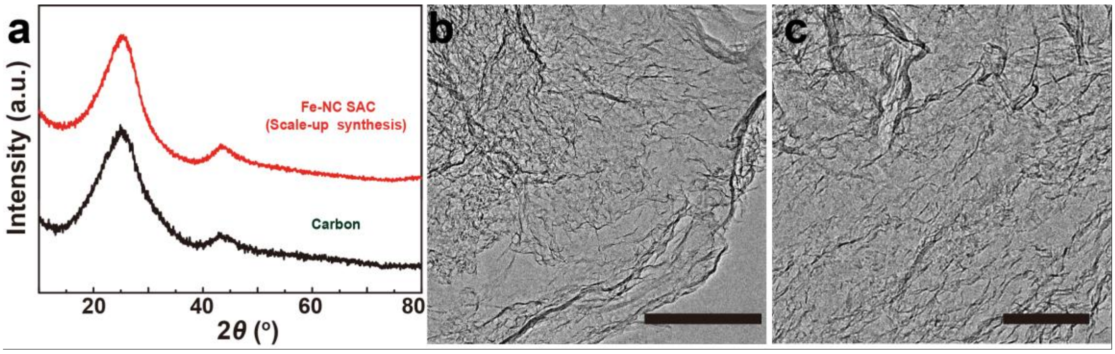

Supplementary Figure 17. a, XRD patterns for carbon substrate and Fe-NC SAC prepared in a scale-up synthesis. **b, c**, TEM images of Fe-NC SAC prepared in a scale-up synthesis. Scale bars, 200 nm (**b**); 100 nm (**c**).

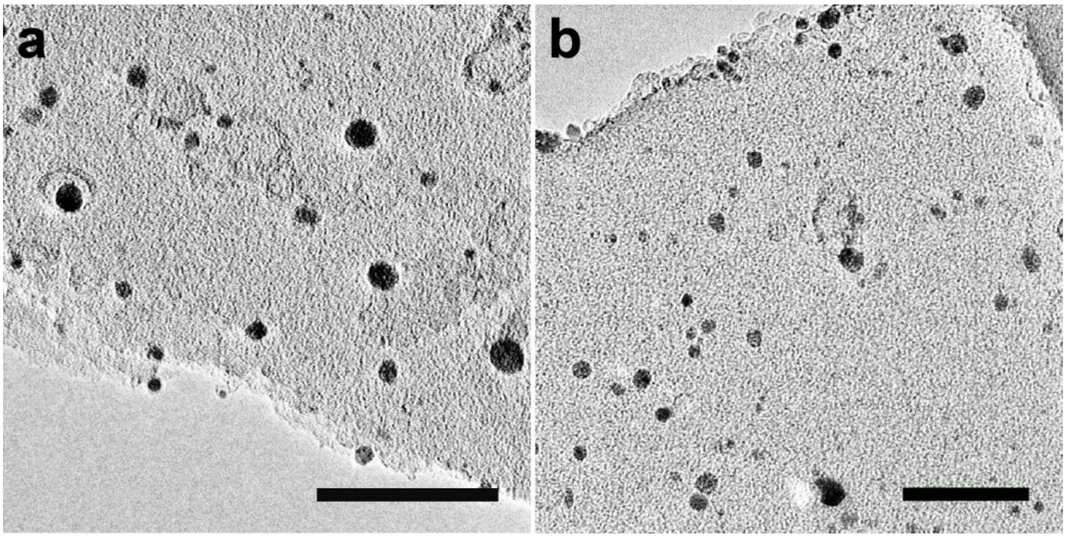

Supplementary Figure 18. a, b, TEM images of control sample Fe@C-N. Scale bars, 100 nm (**a**, **b**).

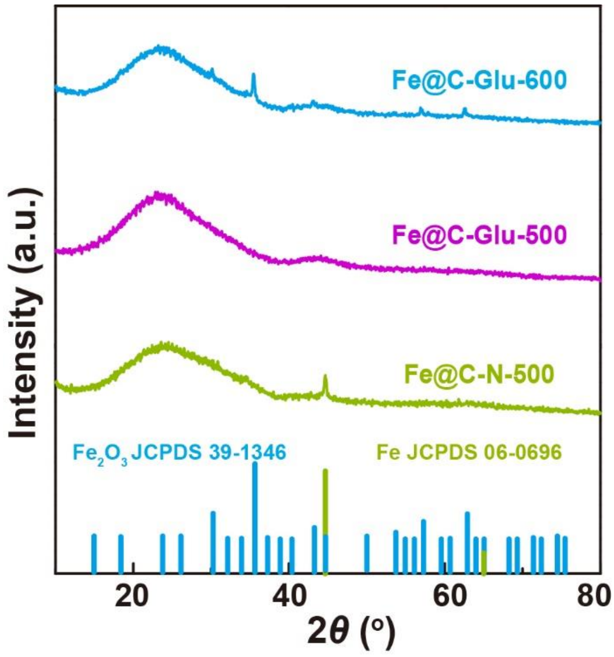

Supplementary Figure 19. XRD patterns of control sample Fe@C-N-500, Fe@C-Glu-500, and Fe@C-Glu-600.

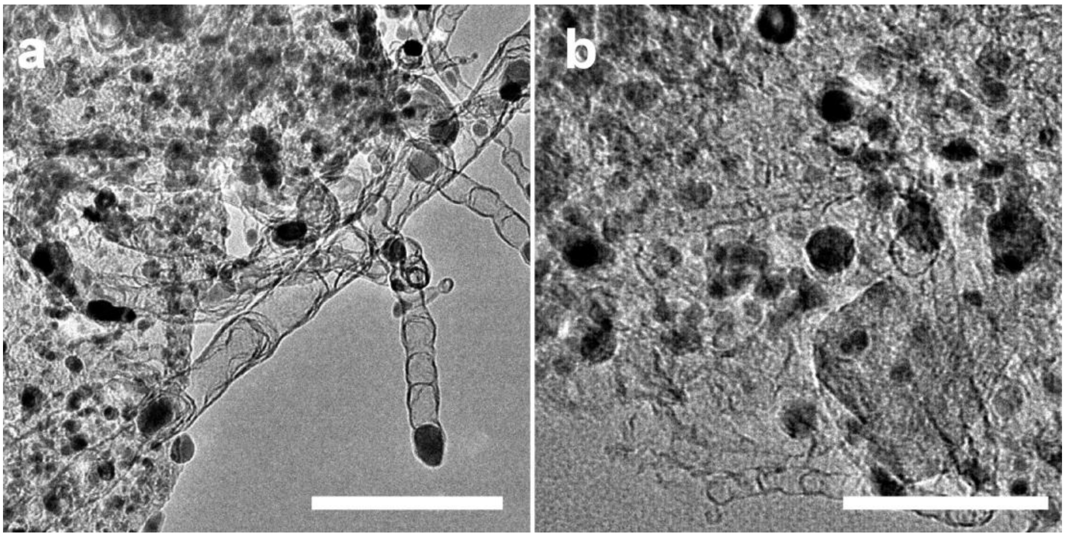

Supplementary Figure 20. a, **b**, TEM images of control sample $Fe(acac)_3$-NC. Scale bars, 200 nm (**a**); 100 nm (**b**).

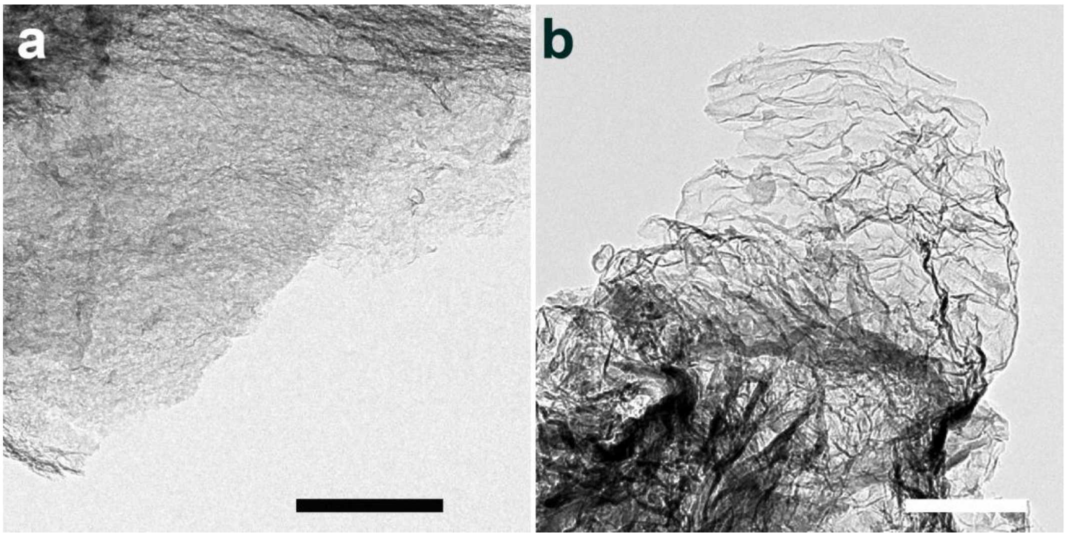

Supplementary Figure 21. a, **b**, TEM images of control sample Fe-NC SAC-EDTA. Scale bars, 300 nm (**a**); 200 nm (**b**).

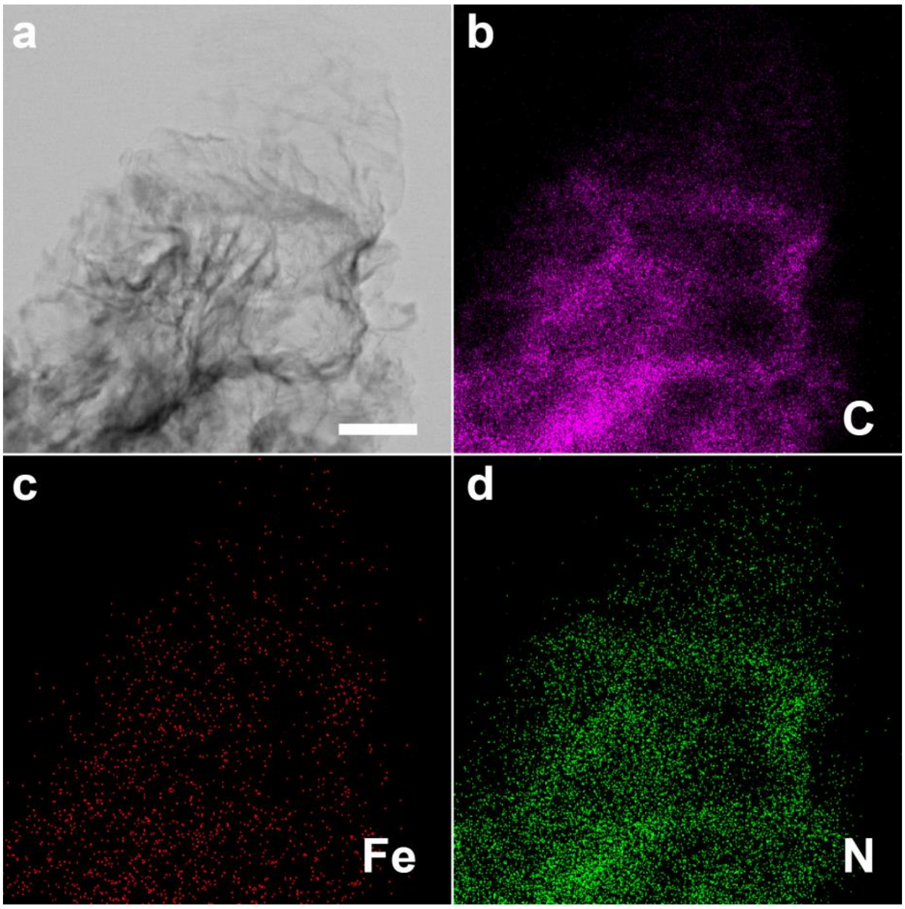

Supplementary Figure 22. a, STEM image, and **b-d**, EDS mapping images of control sample Fe-NC SAC-EDTA: C (**b**), Fe (**c**), and N (**d**). Scale bar, 200 nm (**a**).

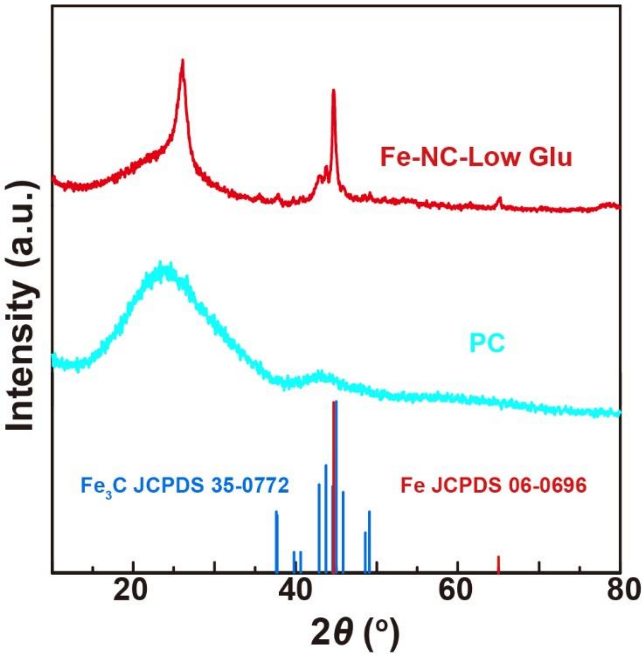

Supplementary Figure 23. XRD patterns of control sample Fe-NC-Low Glu and PC substrate.

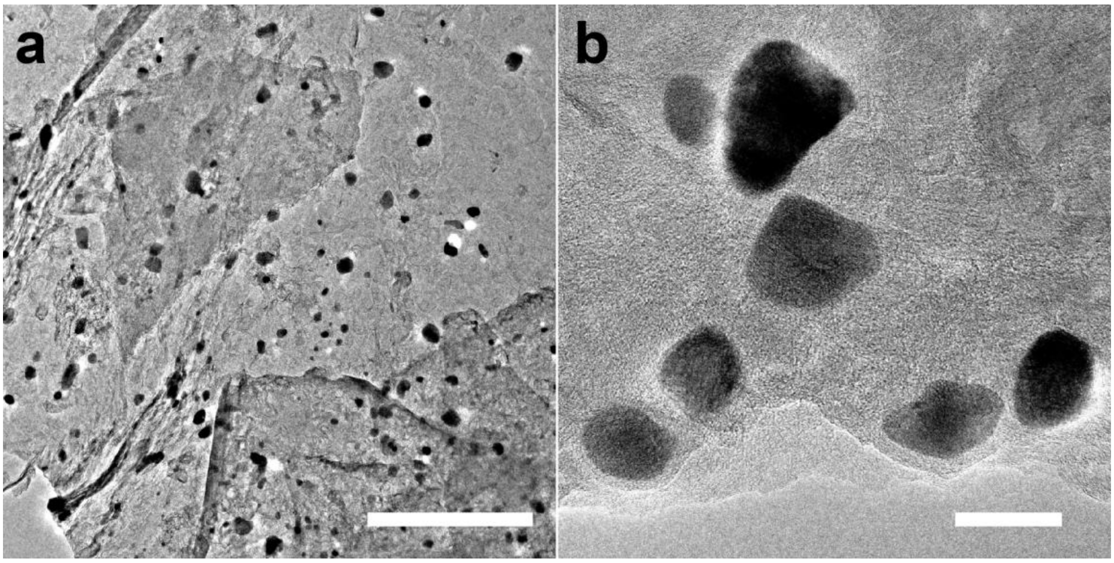

Supplementary Figure 24. a, **b**, TEM images of control sample Fe-NC-Low Glu. Scale bars, 500 nm (**a**); 25 nm (**b**).

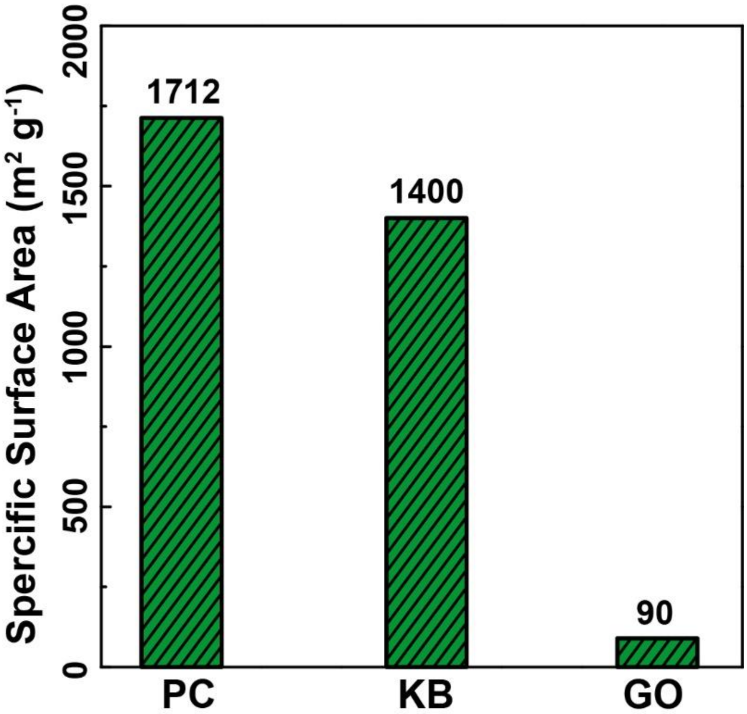

Supplementary Figure 25. Specific surface areas of PC, KB and GO.

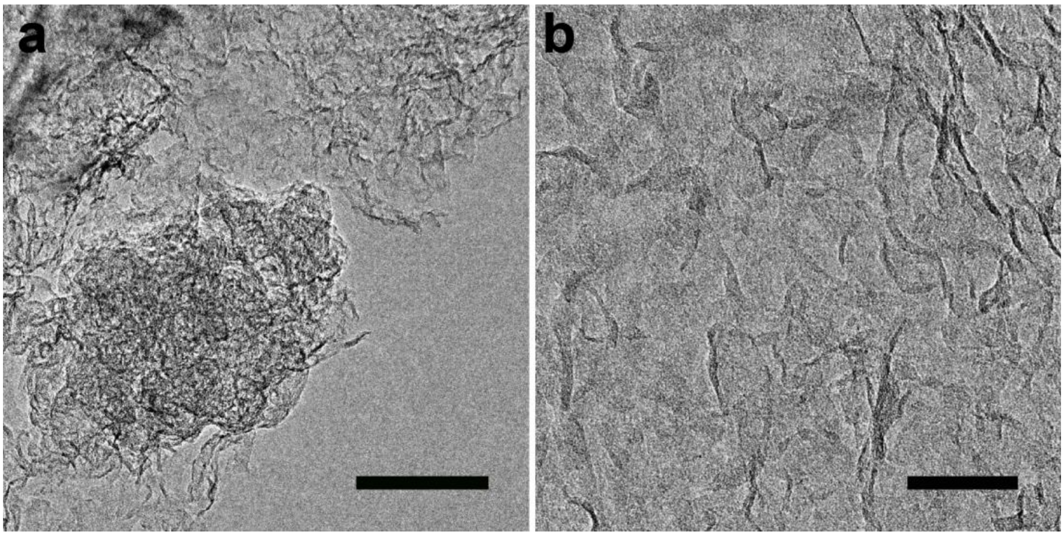

Supplementary Figure 26. **a**, **b**, TEM images of control sample Fe-NC SAC-KB. Scale bars, 100 nm (**a**); 50 nm (**b**).

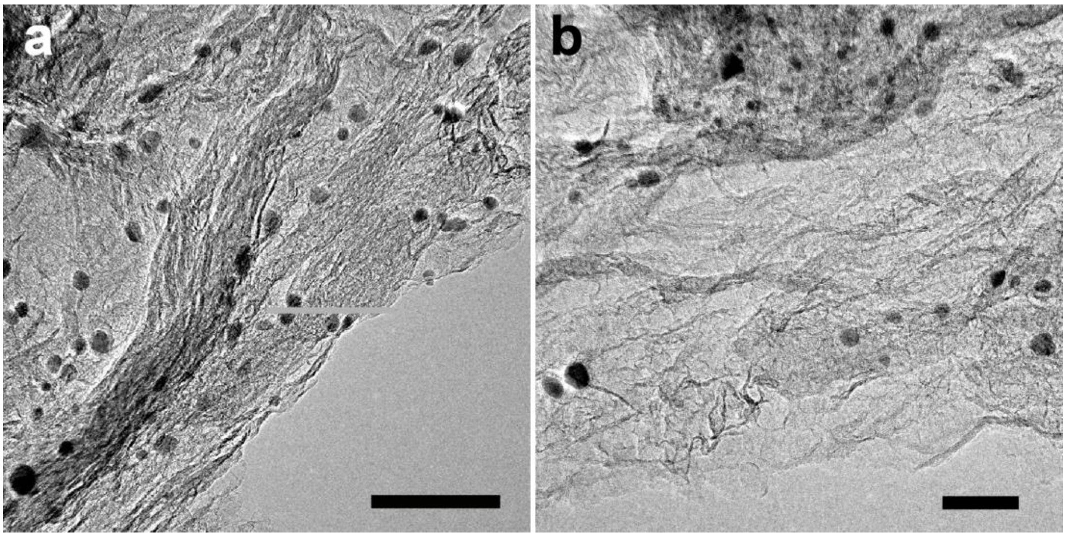

Supplementary Figure 27. **a**, **b**, TEM images of control sample Fe-N-GO. Scale bars, 100 nm (**a**); 50 nm (**b**).

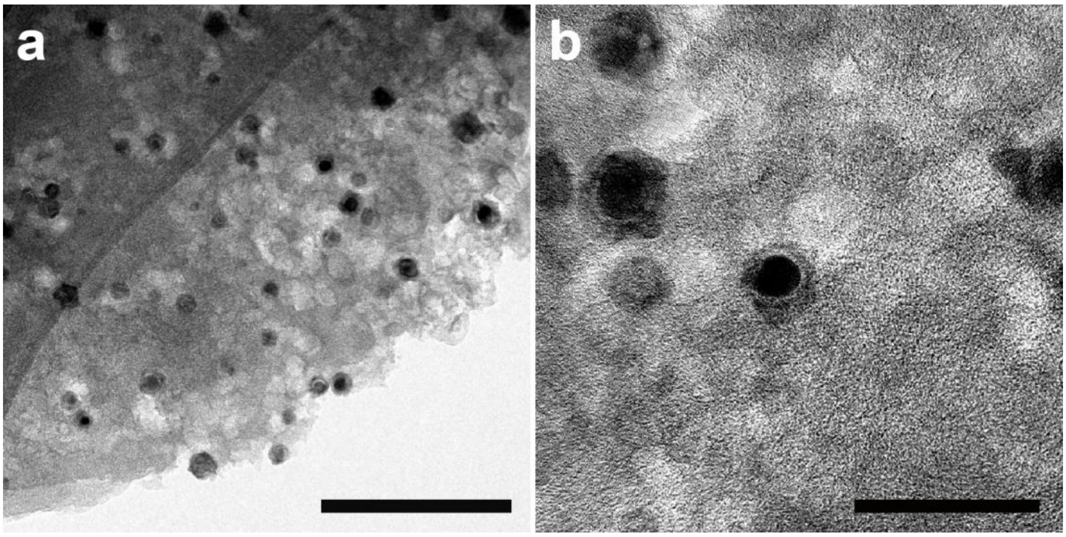

Supplementary Figure 28. a, **b**, TEM images of control sample Fe@C-Glu. Scale bars, 200 nm (**a**); 50 nm (**b**).

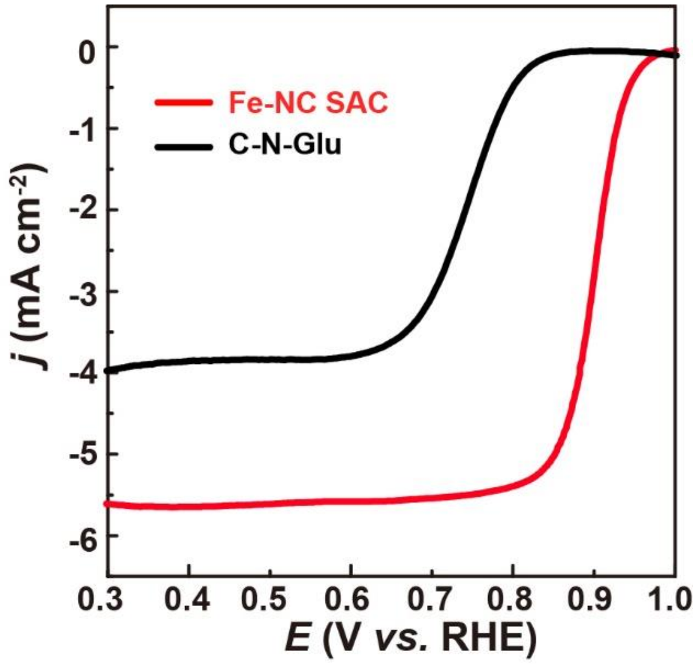

Supplementary Figure 29. Steady-state ORR polarization curves of Fe-NC SAC and control sample C-N-Glu, recorded in 0.1 M KOH.

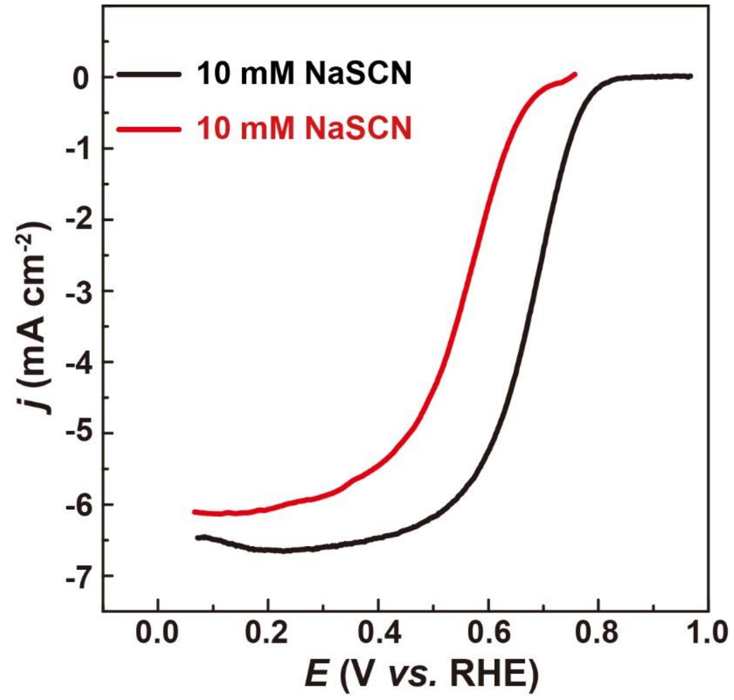

Supplementary Figure 30. Steady-state ORR polarization curves recorded on Fe-NC SAC in O_2-saturated 0.1 M $HClO_4$ with or without 0.01 M SCN^-.

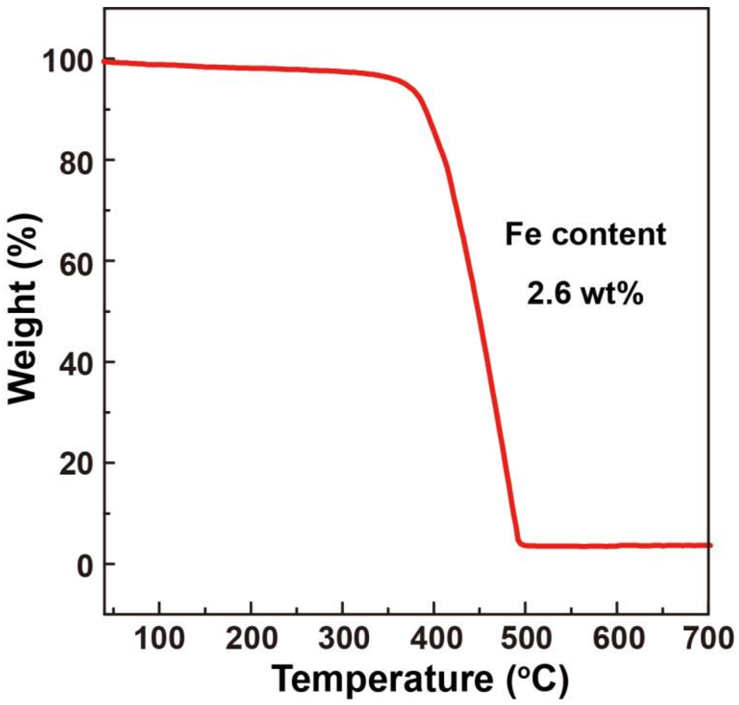

Supplementary Figure 31. TGA curve collected in air atmosphere for Fe-NC SAC with Low Loading.

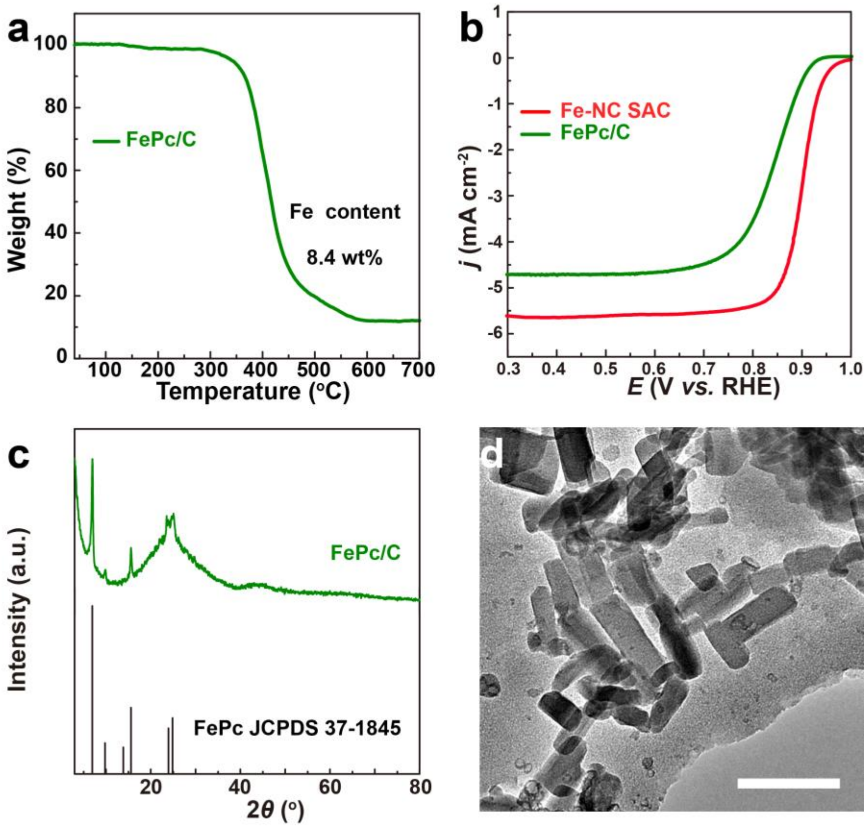

Supplementary Figure 32. **a**, TGA curve collected in air atmosphere for reference FePc/C. **b**, Steady-state ORR polarization curves of Fe-NC SAC and reference FePc/C, recorded in 0.1 M KOH. **c,** XRD pattern and **d,** TEM image for reference FePc/C. Scale bar, 200 nm (**d**).

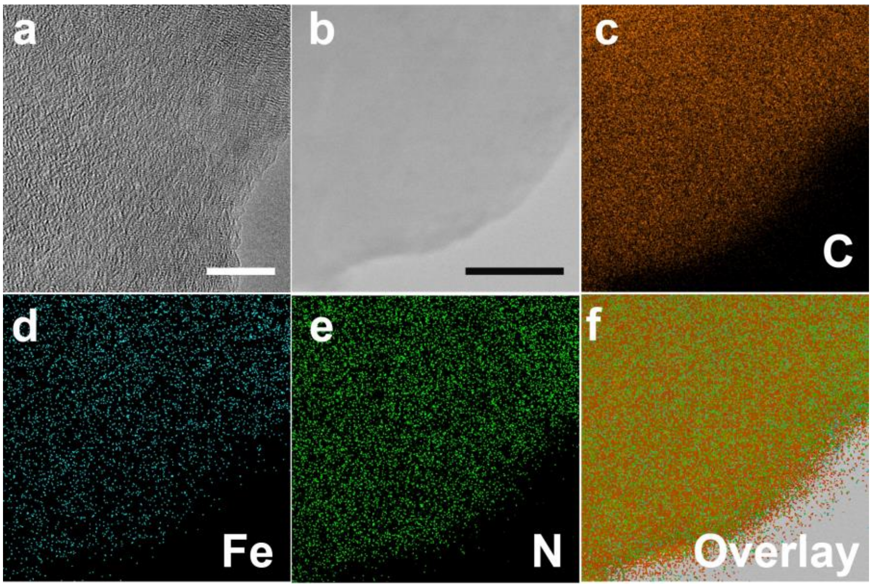

Supplementary Figure 33. **a**, TEM image, **b**, STEM image, and **c-f**, EDS mapping images of Fe-NC SAC after ADT: C (**c**), Fe (**d**), N (**e**), and integrated elemental mapping image (**f**). Scale bars, 10 nm (**a**); 100 nm (**b**).

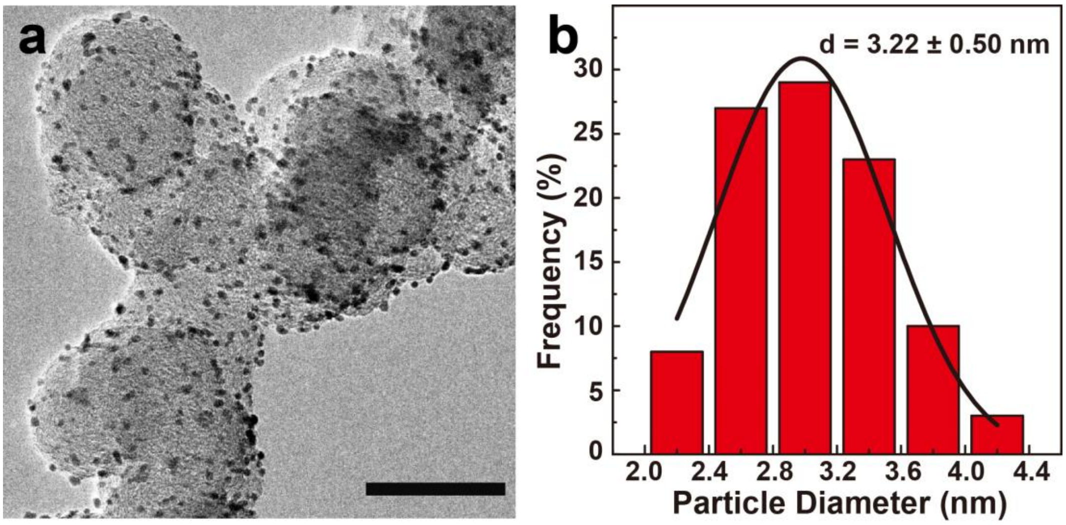

Supplementary Figure 34. **a**, TEM image and **b**, Size distribution of Pt nanoparticles for Johnson-Matthey Pt/C (20 wt%). Scale bar, 50 nm (**a**).

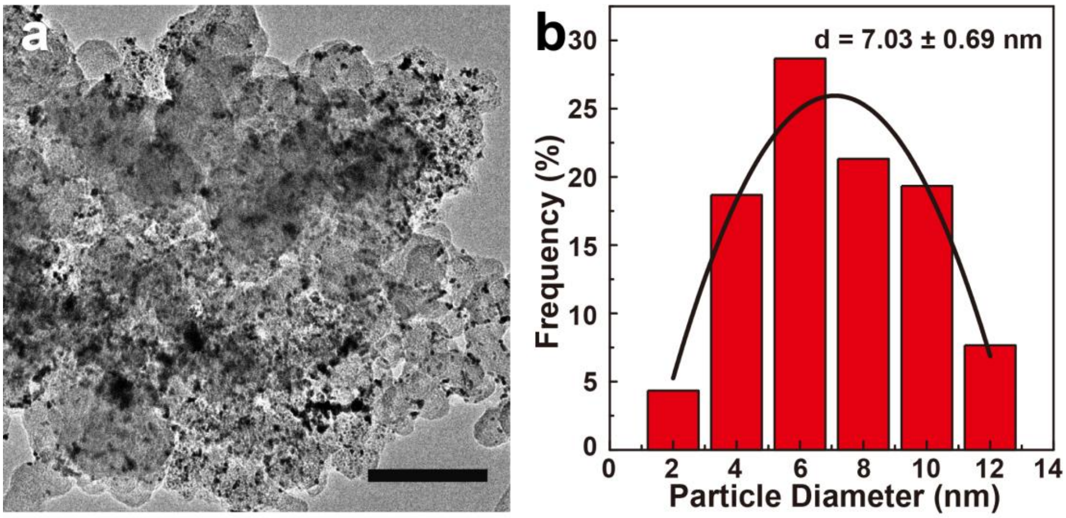

Supplementary Figure 35. **a**, TEM image and **b**, Size distribution of Pt nanoparticles for Johnson-Matthey Pt/C (20 wt%) after ADT. Scale bar, 100 nm (**a**).

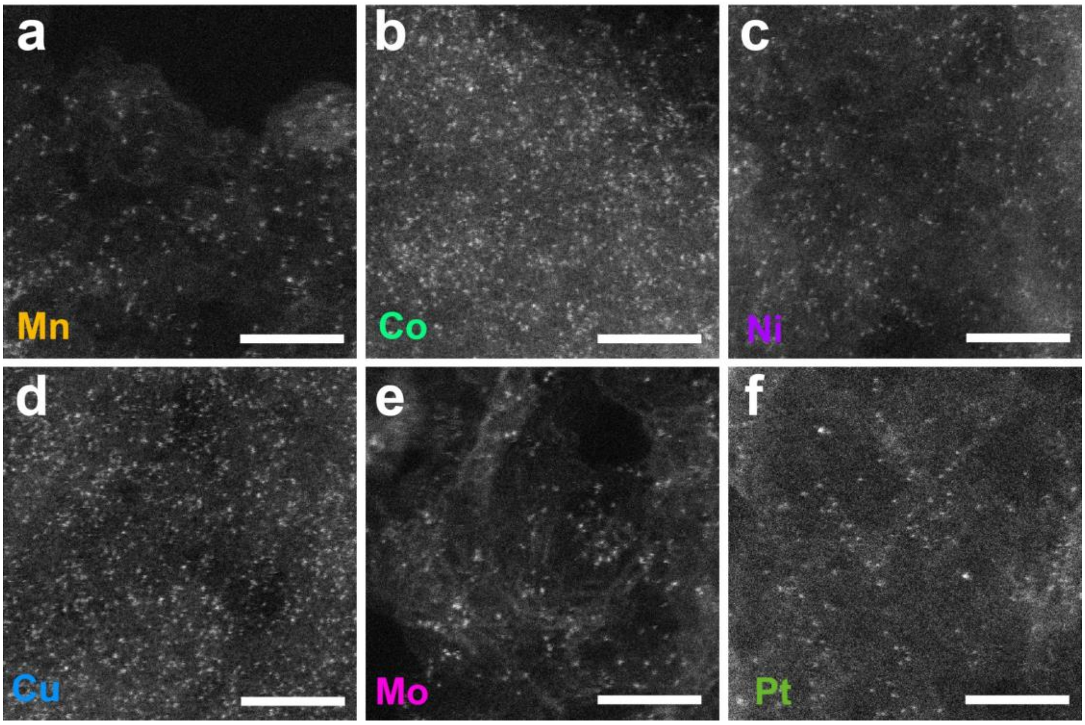

Supplementary Figure 36. a-f, Supplementary HAADF-STEM images for Mn-NC SAC (**a**), Co-NC SAC (**b**), Ni-NC SAC (**c**), Cu-NC SAC (**d**), Mo-NC SAC (**e**), and Pt-NC SAC (**f**). Scale bars, 3 nm (**a**-**f**).

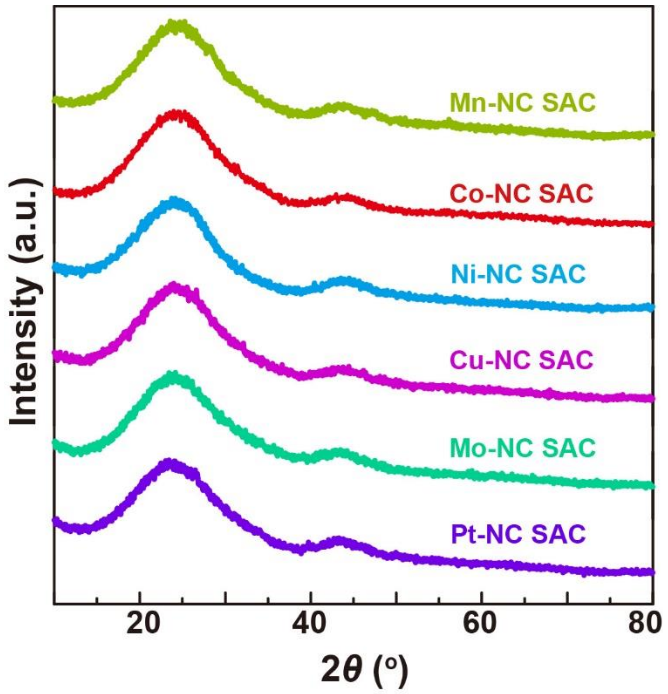

Supplementary Figure 37. XRD patterns of Mn-NC SAC, Co-NC SAC, Ni-NC SAC, Cu-NC SAC, Mo-NC SAC, and Pt-NC SAC.

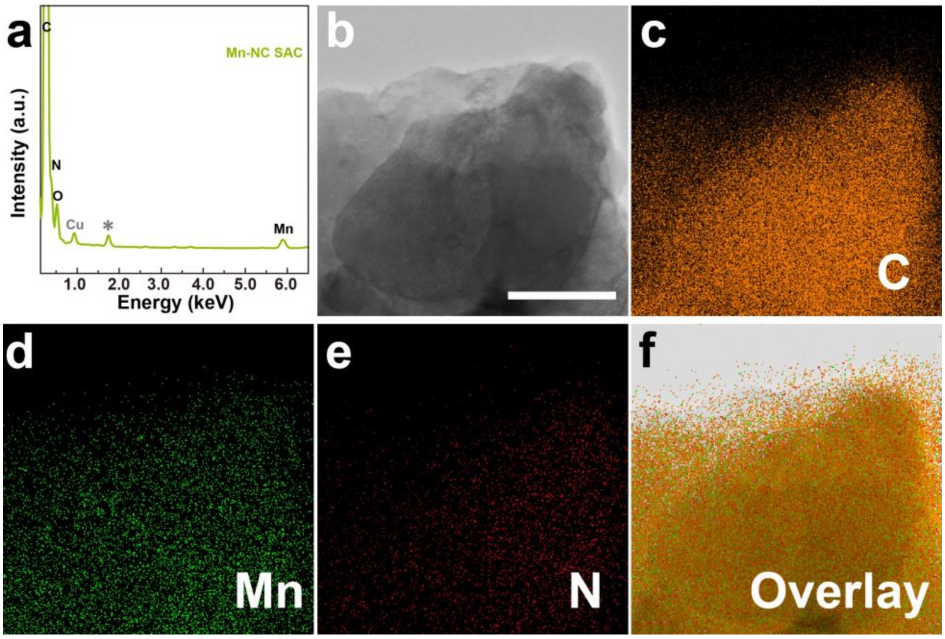

Supplementary Figure 38. a, EDS spectrum, **b**, STEM image, and **c-f**, EDS mapping images of Mn-NC SAC: C (**c**), Mn (**d**), N (**e**), and integrated elemental mapping image (**f**). The signals of Cu and one marked by grey star in the spectrum come from TEM Cu grid (as indicated by EDS spectrum of blank TEM Cu grid in Supplementary **Fig. 44a**). Scale bar, 250 nm (**b**).

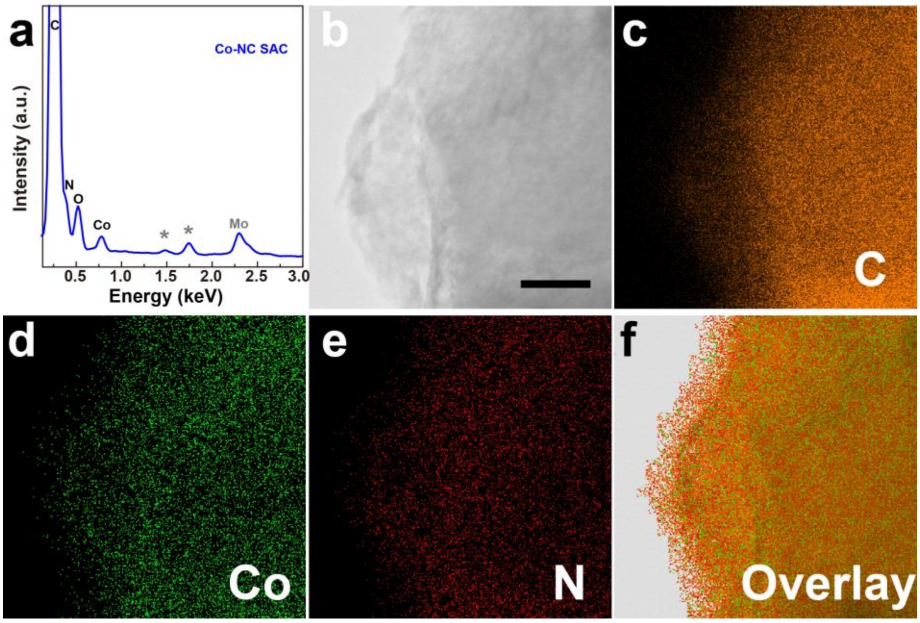

Supplementary Figure 39. a, EDS spectrum, **b**, STEM image, and **c-f**, EDS mapping images of Co-NC SAC: C (**c**), Co (**d**), N (**e**), and integrated elemental mapping image (**f**). The signals of Mo and ones marked by grey stars in the spectrum come from TEM Mo grid (as indicated by EDS spectrum of blank TEM Mo grid (Supplementary **Fig. 44b**). Scale bar, 100 nm (**b**).

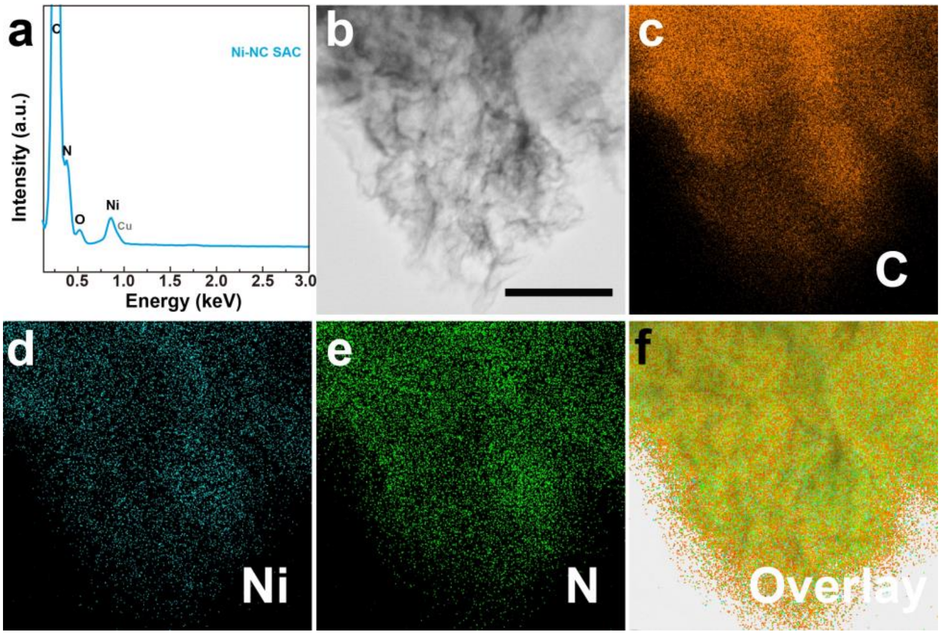

Supplementary Figure 40. **a**, EDS spectrum, **b**, STEM image, and **c-f**, EDS mapping images of Ni-NC SAC: C (**c**), Ni (**d**), N (**e**), and integrated elemental mapping image (**f**). The weak signal of Cu in the spectrum comes from TEM Cu grid (as indicated by EDS spectrum of blank TEM Cu grid in Supplementary **Fig. 44a**). Scale bar, 200 nm (**b**).

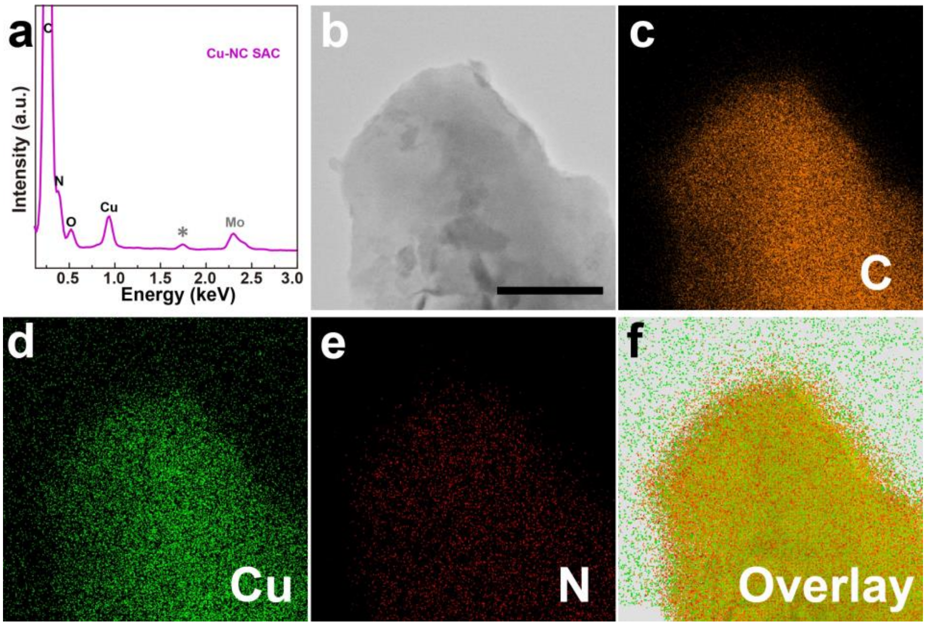

Supplementary Figure 41. **a**, EDS spectrum, **b**, STEM image, and **c-f**, EDS mapping images of Cu-NC SAC: C (**c**), Cu (**d**), N (**e**), and integrated elemental mapping image (**f**). The signals of Mo and one marked by grey star in the spectrum come from TEM Mo grid (as indicated by EDS spectrum of blank TEM Mo grid (Supplementary **Fig. 44b**). Scale bar, 250 nm (**b**).

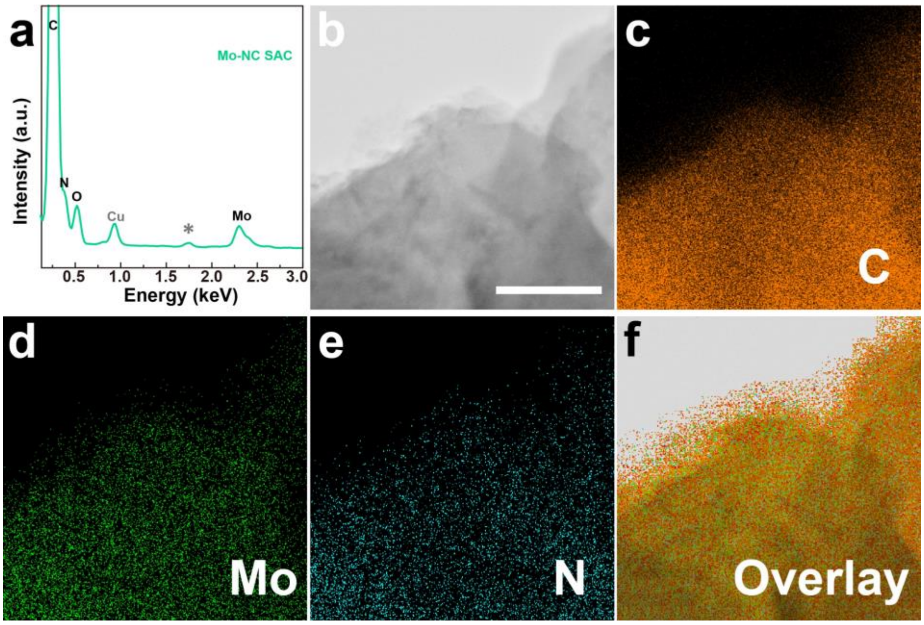

Supplementary Figure 42. a, EDS spectrum, **b**, STEM image, and **c-f**, EDS mapping images of Mo-NC SAC: C (**c**), Mo (**d**), N (**e**), and integrated elemental mapping image (**f**). The signals of Cu and one marked by grey star in the spectrum come from TEM Cu grid (as indicated by EDS spectrum of blank TEM Cu grid (Supplementary **Fig. 44a**). Scale bar, 200 nm (**b**).

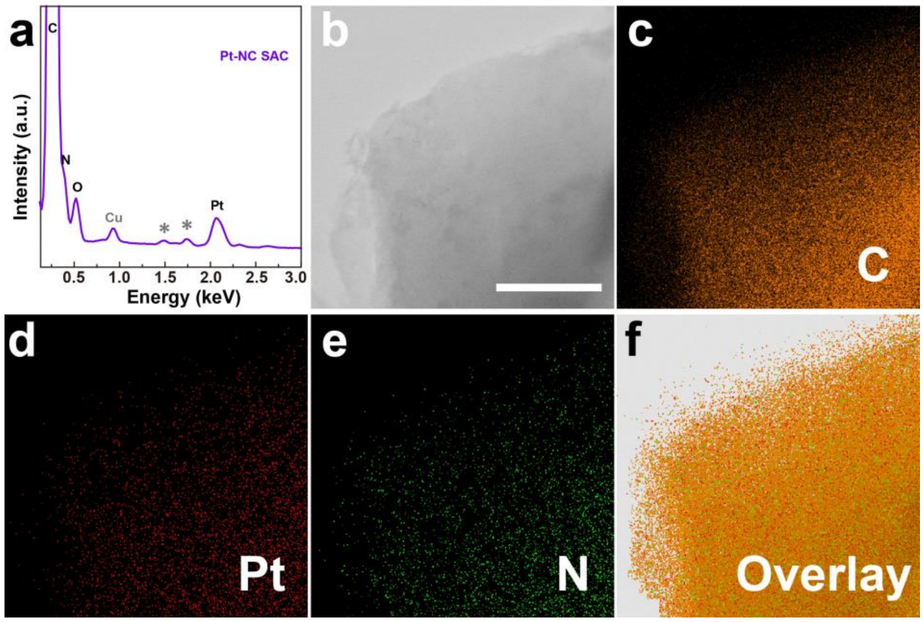

Supplementary Figure 43. a, EDS spectrum, **b**, STEM image, and **c-f**, EDS mapping images of Pt-NC SAC: C (**c**), Pt (**d**), N (**e**), and integrated elemental mapping image (**f**). The signals of Cu and ones marked by grey stars in the spectrum come from TEM Cu grid (as indicated by EDS spectrum of blank TEM Cu grid (Supplementary **Fig. 44a**). Scale bar, 200 nm (**b**).

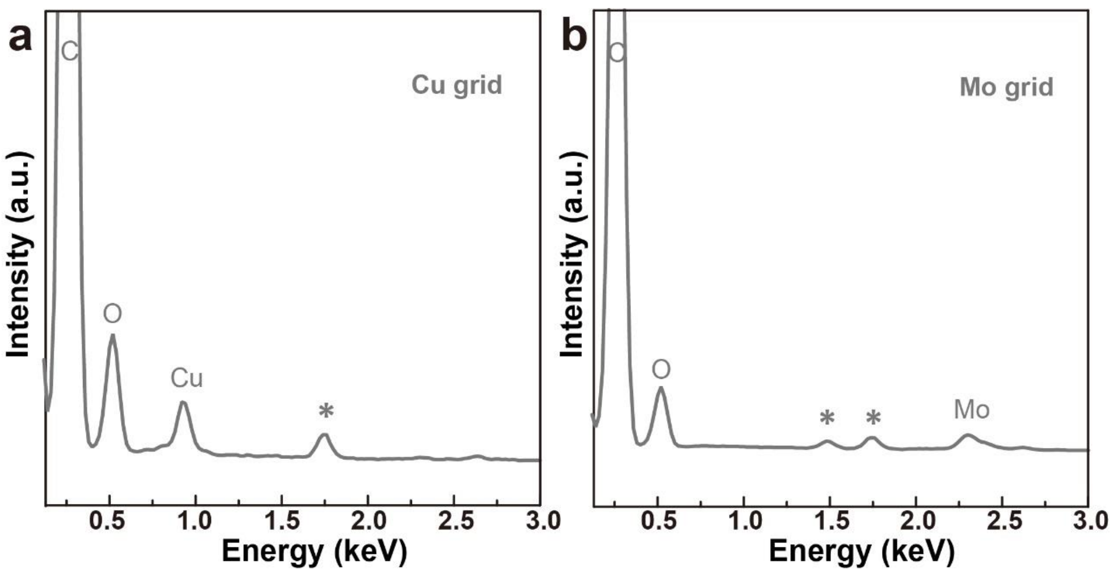

Supplementary Figure 44. a, **b**, EDS spectra of blank TEM Cu (**a**) and Mo (**b**) grid.

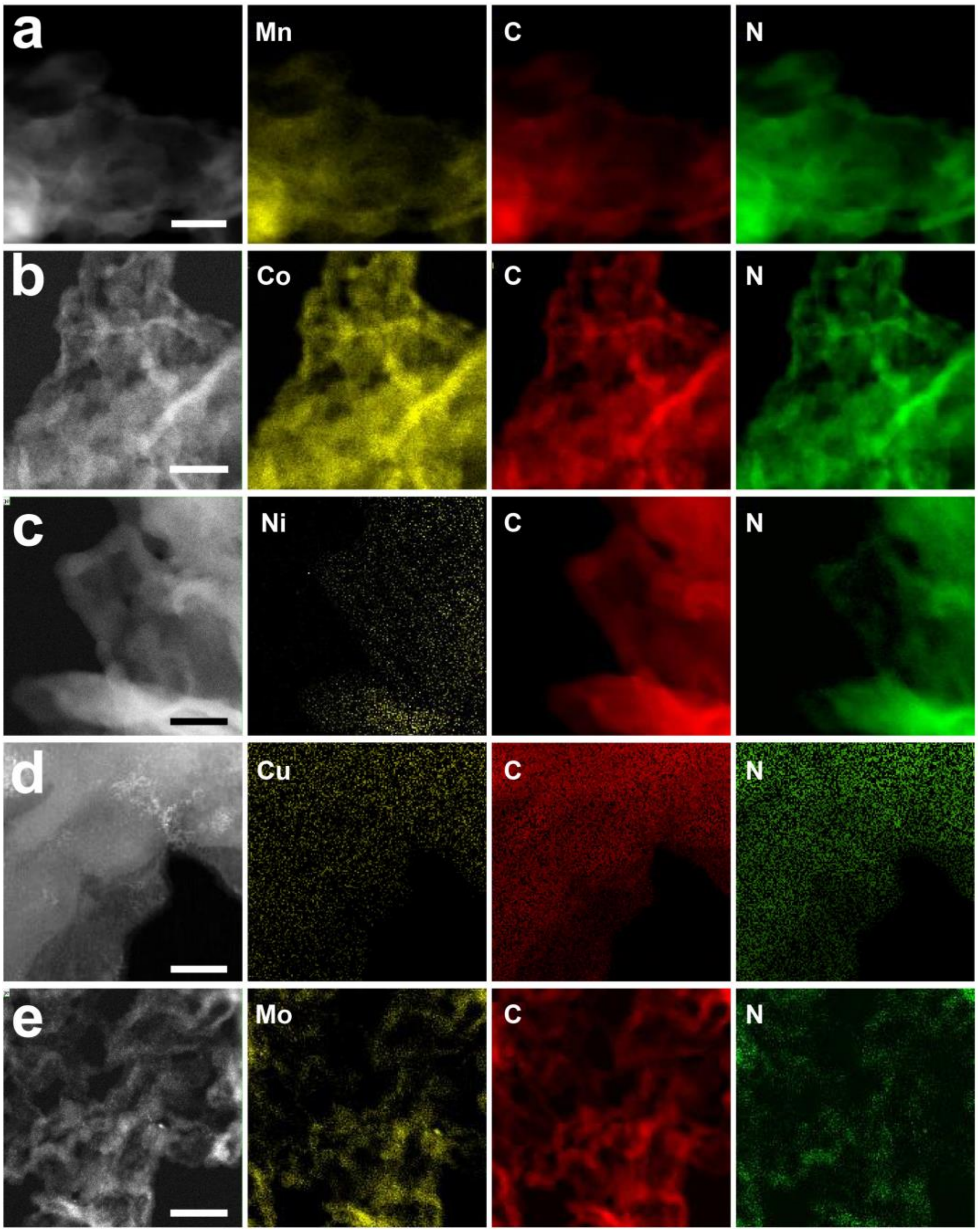

Supplementary Figure 45. a-e, HAADF-STEM and EELS mapping images of Mn-NC SAC (**a**), Co-NC SAC (**b**), Ni-NC SAC(**c**), Cu-NC SAC (**d**), and Mo-NC SAC (**e**). It should be noted that the EELS signal of Pt in Pt-based single-atomic sample is still too weak to have a good image since the characteristic peak of Pt is at very high binding energy (2000 eV) and Pt content is still relatively low. Scale bars, 20 nm (**a**-**e**).

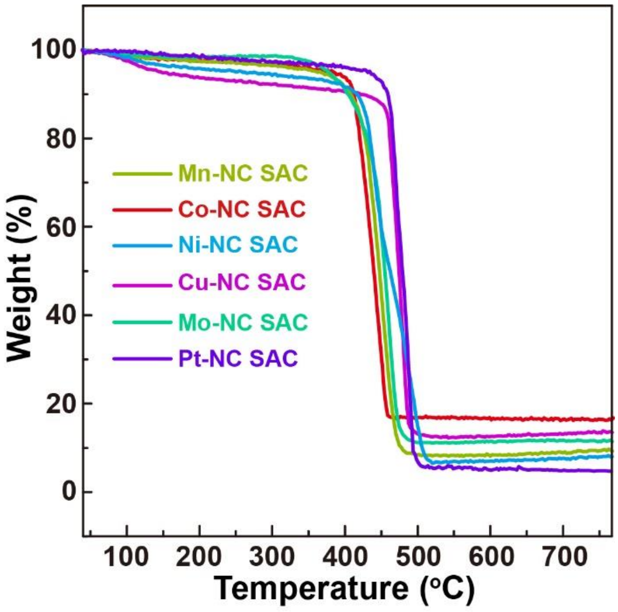

Supplementary Figure 46. TGA analyses in air atmosphere for various M-NC SACs.

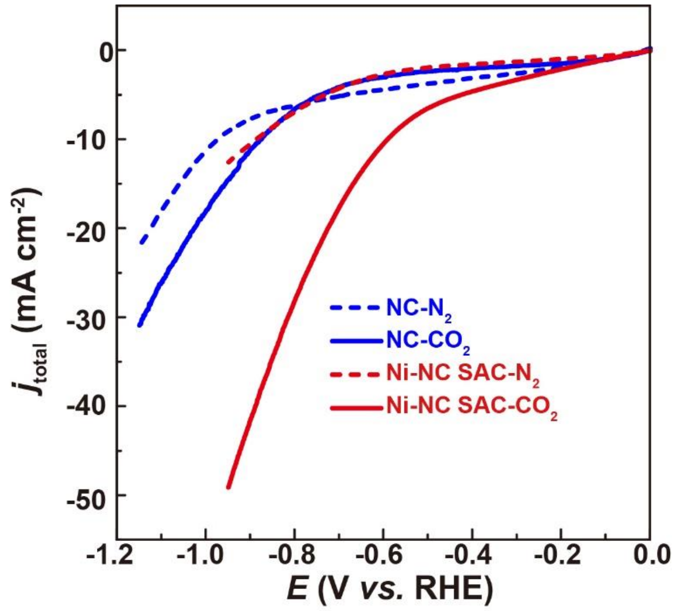

Supplementary Figure 47. Steady-state polarization curves in N_2 and CO_2 saturated 0.5 M $KHCO_3$ solution for Ni-NC SAC and reference sample (NC).

Supplementary Table 1. Parameters of EXAFS fittings for Fe-NC SAC and reference samples (FePc and Fe foil).

Sample	Bond type	Coordination Number	Bond length R(Å)	Bond disorder $\sigma^2 \times 10^{-3}$(Å^2)
**Fe-foil**	Fe-Fe	12	2.52±0.02	8.9±0.8
**FePc**	Fe-N	3.8±0.7	1.93±0.02	2.4±0.6
	Fe-C (second shell)	8.9±0.9	3.00±0.02	6.0±0.9
**Fe-NC SAC**	Fe-N	4.3±0.9	1.99±0.02	7.9±1.0

K-range: 3-12 Å^{-1}; R factor ≤0.02

Supplementary Table 2. Metal content of recently-reported representative single-atom catalysts.

Sample	Metal content	Reference
**Fe-NC SAC**	**8.9 wt% (Fe)**<br>**1.09 at% (Fe)**	**This Work**
**Co-NC SAC**	**12.1 wt% (Co)**	**This Work**
Pt/TiN	0.35 wt% (Pt)	*Angew. Chem. Int. Ed.* **2016**, 55,2058
Pt/HSC	5 wt% (Pt)	*Nat. Commun.* **2016,** 7, 10922.
Fe-N-CNTAs-5-900	0.09 at% (Fe)	*Small* **2017**, 13, 1603407
Co SAs/N-C	4 wt% (Co)	*Angew. Chem., Int. Ed.* **2016**, 55, 10800
Co-C_3N_4/CNT	0.2 at% (Co)	*J. Am. Chem. Soc.* **2017**, 139, 3336
Pt_1-N/BP	0.4 wt% (Pt)	*Nat. Commun.* **2017**, 8,15938
Fe-ISAs/CN	2.16 wt% (Fe)	*Angew. Chem. Int. Ed.* **2017**, 56,6937
Co-NG	0.57 at% (Co)	*Nat. Commun.* **2015**, 6, 8668
A-Ni–C	1.5 wt% (Ni)	*Nat. Commun.* **2016**, 7, 10667

Pt-CN	0.16 wt% (Pt)	*Adv. Mater.* **2016**, 28,2427
Au/C	1 wt% (Au)	*Science* **2017**, 355, 1399
Rh_1/ZnO-nw	0.03% wt% (Rh)	*Angew. Chem. Int. Ed.* **2016**, 55, 16054
ISAS-Co/HNCS	2.2 wt% (Co)	*J. Am. Chem. Soc.* **2017**, 139, 17269
Pd_1/TiO_2	1.5 wt% (Pd)	*Science* **2016**, 352,797
Pt/FeO_x-SAC	0.08 wt% (Pt)	*Adv. Mater.* **2014**, 26, 8147
PtSA-NT-NF	1.76 wt% (Pt)	*Angew. Chem. Int. Ed.* **2017**,56,13694
Pt_1/FeO_x	0.17 wt% (Pt)	*Nat. Chem.* **2011**, 3, 63
Pt-PMA/AC	0.91 wt% (Pt)	*Angew. Chem. Int. Ed.* **2016**, 55, 8319
Co-N/CNFs	0.66 at% (Co)	*ACS Catal.* **2017**, 7, 6864
FeN_4/GN-2.7	2.7 wt% (Fe)	Nano Energy **2017**, 32, 353
Co-N-C	3.6 wt% (Co)	*Chem. Sci.* **2016**, 7, 5758
Co-ISAS/p-CN	0.42 wt% (Co)	*Adv. Mater.* **2018,** 30, 1706508
M-NHGFs (M = Fe, Co, Ni)	~0.05 at% (M)	*Nat. Catal.* **2018**, 1, 63
Ni-NG	0.44 at% (Ni)	*Energy Environ. Sci.* **2018**, 11, 893
Co_1-G	1.2 wt% (Co)	*Adv. Mater.* **2018**, 30, 1704624
Al-TCPP-0.3Pt	0.29 wt% (Pt)	*Adv. Mater.* **2018**, 30, 1705112

Supplementary Table 3. ORR performance in 0.1 M KOH of the catalysts we prepared in this manuscript.

Sample	Onset potential (V *vs.* RHE)	Half wave potential (V *vs.* RHE)
**Fe-NC SAC**	**0.98**	**0.90**
Fe@C-Glu	0.81	0.68
Fe@C-N	0.95	0.78
C-N-Glu	0.85	0.74
FePc/C	0.93	0.94
Pt/C (20 wt%)	0.96	0.85
Fe-NC SAC (2.6 wt%)	0.92	0.82

Supplementary Table 4. ORR performance comparison for typical non-precious-metal electrocatalysts reported in recent years[§].

Sample	E_{onset}* (V *vs.* RHE)	$E_{1/2}$ (V *vs.* RHE)	Tafel slope (mV dec^{-1})	*n*	TOF (e $site^{-1}$ s^{-1})	Reference
**Fe-NC SAC**	**0.98**	**0.90**	**48**	**4.0**	**0.06** (0.9 V)	**This work**
(CM+PANI)-Fe-C (in 0.5 M H_2SO_4)	--	0.80	--	>3.95	--	*Science* **2017**, 357, 479
PANI-Fe-C (in 0.5 M H_2SO_4)	0.91-0.93	0.81	87	--	--	*Science* **2011**, 332, 443
N-Fe-CNT/CNP (in 0.1 M NaOH)	--	0.93	79	--	--	*Nat. Commun.* **2013**, 4, 1922
CNT/PC(FeNC)	--	0.88	--	--	1.7 [a] (0.8 V)	*J. Am. Chem. Soc.* **2016**, 138, 15046
FP-Fe-TA-N-850	0.98	--	--	3.5-3.9	3.0 [b] (0.8 V)	*Angew. Chem. Int. Ed* **2016**, 55, 1355
Fe-N-C-2HT-1AL	0.92	0.82	--	--	0.7 [c] (0.8 V)	*Nat. Commun.* **2015**, 6, 8618
Fe-N-C-3HT-2AL	0.94	0.85	--	--	1.7 [c] (0.8 V)	
Fe@Aza-PON	--	0.84	60	3.7	--	*J. Am. Chem. Soc.* **2018,** 140, 1737
p-Fe-N-CNFs	0.94	0.82	--	--	--	*Energy Environ. Sci.* **2018**, 11, 2208
FeNC-S-FexC/Fe	1.05	0.873	--	5.45	--	*Adv. Mater.* **2018**, 1804504
GL-Fe/Fe_5C_2/NG-800	0.98	0.86	--	3.95	--	*Adv. Energy Mater.* **2018**, 8, 1702476
FePhen@MOF-$ArNH_3$	1.03	0.86	--	--	--	*Nat. Commun.* **2015**, 6, 7343
Fe2-Z8-C	0.985	0.871	--	--	--	*Angew.Chem.Int.Ed.* **2018**, 57, 1204
Co SAs/N-C(900)	0.98	0.88	75	~4.0	--	*Angew. Chem. Int. Ed.* **2016**, 55, 10800
Co,N-CNF	0.88	0.82	60	3.8	--	*Adv. Mater.* **2016**, 28, 1668
NC@Co-NGC DSNCs	0.92	0.82	51	~4.0	--	*Adv. Mater.* **2017**, 29, 1700874
PMF-800	0.92	0.86	91.2	3.99	--	*J. Am. Chem. Soc.* **2015**, 137,1436
h-Mn_3O_4-TMSLs	0.91	0.84	71	3.91	--	*J. Am. Chem. Soc.* **2017**, 139, 12133

CoO_x NPs/BNG	0.95	0.81	--	4.0	--	*Angew. Chem. Int. Ed.* **2017**, 56, 7121
NCNTFs	0.97	0.87	64	3.97	--	*Nat. Energy* **2016**, 1, 15006
NBCFM/N-rGO	--	0.889	44	4.0	--	*Sci. Adv.* **2018**, 4, eaap9360

E_{onset}: onset potential; $E_{1/2}$: half-wave potential; *n*: electron transfer number.

[§] These data were measured in 0.1 M KOH unless specified.

[*] The reported onset-potentials are based on different definitions.

[a] The Fe content was obtained from Mössbauer spectroscopy.

[b] The Fe content was calculated from the XPS data.

[c] The Fe content was based on the accessible surface sites probed by CO chemisorption.

The electron transfer number *n* is calculated by the following equation:

$$n = 4 \times \frac{I_D}{(I_R/N) + I_D} \quad (4)$$

Where I_D and I_R are the disk and ring currents, respectively; *N* is the ring collection efficiency and equals to 0.424.

The TOF is estimated according to the reported method[4-5]. In our developed Fe-NC SAC, the ORR activity should be mainly ascribed to Fe-N_x in single-atomic sate. Therefore, by assuming that all of Fe atoms (8.9 wt%) could contribute to ORR activity (which may underestimate the TOF of our catalyst since some of Fe may not be accessible), TOF of Fe-NC SAC can be calculates as follows:

The density of active site is:

$$D = \frac{w_{Fe}}{M_{Fe}} \times N_A = \frac{0.089}{55.85} \times 6.02 \times 10^{23} = 9.59 \times 10^{20} \text{ site/g} \quad (5)$$

Where M_{Fe} is the molar mass of iron (55.85 g/mol), N_A is Avogadro's number (6.02×10^{23}).

The kinetic current density at 0.9 V ($j_{k,\ 0.9\ V}$) is calculated from K-L equation:

$$j_{k,\ 0.9\ V} = \frac{j_D \times j_{0.9\ V}}{j_D - j_{0.9\ V}} = \frac{5.58 \times 2.74}{5.58 - 2.74} = 5.38 \text{ mA/cm}^2 \quad (6)$$

The j_D *and* $j_{0.9\ V}$ were read from LSV curves at the rotating speed of 1600 rpm for Fe-NC SAC (m_{cat}; catalyst loading: 0.6 mg/cm^2).

Then kinetic current density normalized to mass of catalyst at 0.9 V ($j_{k,\ 0.9\ V,\ m}$):

$$j_{k,\ 0.9\ V,\ m} = \frac{j_{k,\ 0.9\ V}}{m_{cat}} = \frac{5.38}{0.6} = 8.97\ A/g \qquad (7)$$

Then TOF at 0.9 V is:

$$TOF = \frac{j_{k,\ 0.9\ V}}{e \times D} = \frac{8.97}{1.6 \times 10^{-19} \times 9.59 \times 10^{20}} = 0.06\ e\ sit^{-1}s^{-1} \qquad (8)$$

It should be noted that TOF value is strongly dependent on the used potential and the density of active sites. In different reports, the density of active sites was determined by different characterization methods, such as XPS data, CO chemisorption content; doublet percent in Mössbauer spectroscopy, et al[6-8]. These methods give the surface composition to calculate TOF. For a metal-based catalyst, the metal atoms in the bulk such as nanoparticles may not be counted on. In our calculation for Fe-NC SAC, we used TGA analysis to determine the total metal content and counted all Fe atoms.

Moreover, TOF values should be calculated and reported at a specific potential. In most of reports, they reported TOF at 0.8 V. In our case, j_L is already limited by diffusion at 0.8 V, causing the improper evaluation. Therefore, we calculated TOF at 0.9 V.

Supplementary Table 5. Parameters for electrocatalytic CO_2 reduction to CO on Ni-NC SAC and reference sample NC.

Potential (V *vs.* RHE)		-0.55	-0.65	-0.75	-0.8	-0.85	-0.95
CO (%)	NC	9.7	10.3	12.8	8.7	5.8	3.2
	Ni-NC SAC	60.7	77.8	83.2	88.2	89.0	84.8
j_{CO} (mA cm^{-2})	NC	0.1	0.4	0.6	0.6	0.6	0.7
	Ni-NC SAC	4.0	9.4	15.9	26.0	30.0	36.7

Supplementary Table 6. Performance comparison for electrocatalytic CO_2 reduction to CO in $KHCO_3$ condition for our Ni-NC SAC and other SACs reported recently.

Sample	Overpotential (V *vs.* RHE)	Faradaic efficiency (%)	Current density (CO, peak potential) (mA cm^{-2})	Reference
**Ni-NC SAC**	**-0.74**	**89**	**30.0**	**This work**
Ni SAs/N-C	-0.89	72	10.5	*J. Am. Chem. Soc.* **2017**, 139, 8078
A-Ni-NSG	-0.61	94	22.1	*Nat. Energy* **2018**, 3, 140
Ni-N-C	-0.64	96	8.2	*Appl. Catal., B* **2018**, 226, 463
Ni-NG	-0.62	95	10.5	*Energy Environ. Sci.,* **2018**, 11, 893

Reference

1. Nagy, L. et al. Iron(III) complexes of sugar-type ligands. *Inorg. Chim. Acta* **124**, 55-59 (1986).

2. Persson, I. Hydrated metal ions in aqueous solution: How regular are their structures? *Pure Appl. Chem.* **82**, 1901-1917(2010).

3. Heidar, A. & Tajmir, R. Intereaction of D-glucose with alklain-earth metal ions. Synthesis, specteroscopic, and structural characterization of Mg(II)-and Ca(II)-o-glucose adducts and the effect of metal-ion binding on anomeric configuration of the sugar. *Carbohydr. Res.* **138**, 35-46 (1988).

4. Gasteiger, H. A. & Marković, N. M. Just a dream-or future reality? *Science* **324**, 48-49 (2009).

5. Jaouen, F. & Dodelet, J.-P. Average turn-over frequency of O_2 electro-reduction for Fe/N/C and Co/N/C catalysts in PEFCs. *Electrochim. Acta* **52**, 5975-5984 (2007).

6. Wei, J. et al. A versatile iron–tannin-framework ink coating strategy to fabricate biomass-derived iron carbide/Fe-N-carbon catalysts for efficient oxygen reduction. *Angew. Chem. Int. Ed.* **55**, 1355-1359 (2016).

7. Sahraie, N. R. et al. Quantifying the density and utilization of active sites in non-precious metal oxygen electroreduction catalysts. *Nat. Commun.* **6**, 8618 (2015).

8. Sa, Y. J. et al. A general approach to preferential formation of active Fe–N_x sites in Fe–N/C electrocatalysts for efficient oxygen reduction reaction. *J. Am. Chem. Soc.* **138**, 15046-15056 (2016).
