# Supplementary material for: Cascade anchoring strategy for general mass production of high-loading single-atomic metal-nitrogen catalysts
Source: Nat Commun. 2019 Mar 20;10:1278. doi: 10.1038/s41467-019-09290-y (PMC6426845; doi:10.1038/s41467-019-09290-y)
Supplement: Supplementary file 2 — Peer Review [file 41467_2019_9290_MOESM2_ESM.pdf]

Editorial Note: This manuscript has been previously reviewed at another journal that is not operating a transparent peer review scheme. This document only contains reviewer comments and rebuttal letters for versions considered at *Nature Communications.*

Reviewers' comments:

Reviewer #1 (Remarks to the Author):

In this paper, the authors have illustrated a novel method for the mass production of metal-nitrogen single atoms with high loading on carbon support. They have explored the effect of various synthesis parameters on the structure and catalytic activity of synthesized material towards oxygen reduction reaction in alkaline solution (Fe-NC SAC). The authors have illustrated that Fe-NC single atom catalysts exhibit a higher activity and good stability towards oxygen reduction reaction compared to Pt/C commercial catalyst. Based on these studies they have proposed a mechanism for the formation of single atom catalysts on carbon supports. Furthermore, they have extended their process to synthesize other types of metal-nitrogen single atom catalysts. They have used previously conducted theoretical research to back their assumptions in the synthesis process. This work is very interesting, and the authors have carried out detailed study of many factors in the synthesis process. Thus, this manuscript is accepted for publication with minor revisions.

The authors should address some of the following issues:

1. The authors demonstrate the single atom nature of their synthesized products using atomic resolution TEM. They claim in their optimized condition they can achieve very high loading of single atom deposition. However, the illustrated TEM images show bright spots with different diameters (which it has been claimed to be from overlay of atoms at different levels). This is very hard to accept. Would it be possible to show a size distribution chart of at least the spots shown in the TEM images?

2. The authors have employed EXAFS to confirm the single atom nature of the products. They have compared the XAFS data to FePc structure. There are still contradictory reports on the EXAFS modeling of this type of compound. Furthermore, if we accept the model provided for FePc in this manuscript and assuming we have 8% Fe loading in the samples, why can't we see the second carbon shell similar to the FeC in the single atom samples' EXAFS spectra?

3. Comparing the wavelet transform of the Fe, FePc and the single atoms synthesized, it looks like the synthesized samples have a structure between Fe and FePc so claiming single atom formation based on this data is questionable.

4. Regarding the chemical state of Fe in the single atom products, the authors illustrate a +2 oxidation state. Can the authors comment on the oxidation state of other synthesized single atoms such as Pt using this synthesis process?

5. The added text to the original manuscript require further polishing in terms of grammar and wording.

Reviewer #2 (Remarks to the Author):

I am happy with how the authors addressed most of the comments/concerns regarding the manuscript.

However there are still 2 comments where I cannot find an agreement with the interpretation of the authors:

Comment 4: arguing that "a couple of spots shows the size slightly larger than single atom, which could be ascribed to the overlapping of atoms on the different depth profiles of carbon substrate or the rarely-seen atom cluster." is not a clear reflection of what the data show.

using such a highly spatially resolved technique such as HAADF STEM imaging comes with the drawback that the depth resolution is quite limited. This means that if the atoms would be overlapping but on different "depth profiles" then they would need to be very close to each other-if they would be a few nm appart (z-direction) then this should lead to "blurring" and only one of the two imaged atoms would appear sharp.

I think the phrase "rarely seen atom clusters" needs to be removed as the data in Figure 6 clearly indicate the presence of a relatively high amount of clusters in at least b, e and f.

Comment 5: "the pixel size is set during the experiments and should not correlate to the atom size"
if you do not set the pixel size to the size of the atom but much bigger it is not possible to conclude that "N atoms are atomically adjacent to Fe atoms..."

I see that the text has been modified to "imply the possible existence of Fe-N bonding in the sample"; I want to stress again that the experimental EELS maps do not support this statement. Assuming your SI map has a pixel size of about 0.7 nm (scale bar is 2nm and appears to be equivalent to about 3 pixels); this would mean an Fe and N atom could be 4 Angstrom apart and still be measured in the same "pixel".

It is important that these two statements are modified in the manuscript before publication.

Responses to the comments from the Referees

Reviewer 1

In this paper, the authors have illustrated a novel method for the mass production of metal-nitrogen single atoms with high loading on carbon support. They have explored the effect of various synthesis parameters on the structure and catalytic activity of synthesized material towards oxygen reduction reaction in alkaline solution (Fe-NC SAC). The authors have illustrated that Fe-NC single atom catalysts exhibit a higher activity and good stability towards oxygen reduction reaction compared to Pt/C commercial catalyst. Based on these studies they have proposed a mechanism for the formation of single atom catalysts on carbon supports. Furthermore, they have extended their process to synthesize other types of metal-nitrogen single atom catalysts. They have used previously conducted theoretical research to back their assumptions in the synthesis process. This work is very interesting, and the authors have carried out detailed study of many factors in the synthesis process. Thus, this manuscript is accepted for publication with minor revisions.

Thank you very much for such good comment!

Comment 1: The authors demonstrate the single atom nature of their synthesized products using atomic resolution TEM. They claim in their optimized condition they can achieve very high loading of single atom deposition. However, the illustrated TEM images show bright spots with different diameters (which it has been claimed to be from overlay of atoms at different levels). This is very hard to accept. Would it be possible to show a size distribution chart of at least the spots shown in the TEM images?

Response: Thank you for the comments. In the previous manuscript, there are some improper description for the bright spots in different diameters. As the state-of-the-art techniques, atomic-resolution HAADF-STEM together with synchrotron-based XAFS are most powerful to investigate single atom catalysts. We use HAADF-STEM to image single-atomic dispersion of metal atoms and EXAFS to confirm that there is no Fe-Fe bonding. It is reasonable to conclude that we have achieved single atom catalysts. As shown in HAADF-STEM images (**Figs.** 2, 6 and Supplementary **Figs.** 8, 36), it can be clearly seen that most of bright spots in each panel are actually in a size of single atom. Some of spots display slightly different diameters. We have discussed this result with the expert in this field, Prof. Lin Gu at Institute of Physics, Chinese Academy of Sciences. This can be ascribed to the following two factors: 1) in view of the 3D structure of carbon support, the single metal atoms on different profiles were imaged at

the same time. It is impossible to have all atoms at the same focusing plane, resulting in that the sizes of a few bright spots on HAADF-STEM images are slightly different; 2) Nowadays, it is still very difficult to achieve or characterize 100 % single metal atom on bulk substrate in all reported methods. It cannot be 100 % sure in current stage that every atom is in individual state although the EXAFS data shows no metal-metal bonding in the sample. The similar phenomenon for TEM images can be observed in quite a lot of recent high-impact literatures about sing atom catalysts, such as *Science 352, 797(2016); Nat. Nanotechnol. 13, 411(2018); Sci. Adv. 1, e1500462(2015); Adv. Mater., 1705112(2018); Nat. Commun. 9,1460(2018) and Nat. Catal. 1, 781(2018)*. However, basis on our results, it should be convincible that an overwhelming majority of metal atoms in our report should be in single state.

As you suggested, we provided a statistical analysis on the size of these spots. Based on statistical analysis of over 400 bright spots (**Fig.** 2e and Supplementary **Fig.** 8), the average size of spots is 1.04 ± 0.35 Å. This result clearly suggests that the Fe atoms in the Fe-NC SACs are mainly in single-dispersion state.

We have revised the discussion in the revision on Page 6 as follows:

The average size of spots is 1.04 ± 0.35 Å on a basis of statistical analysis on over 400 bright spots) (inset in Supplementary Fig. 8a), corroborating these Fe atoms are mainly in single-atomic state.

We have revised the discussion in the revision on Page 14 as follows:

A couple of spots with different size could be ascribed to the metal atom imaged at different focusing plane or possible atom cluster.

The following inset has been added in the Supporting information.

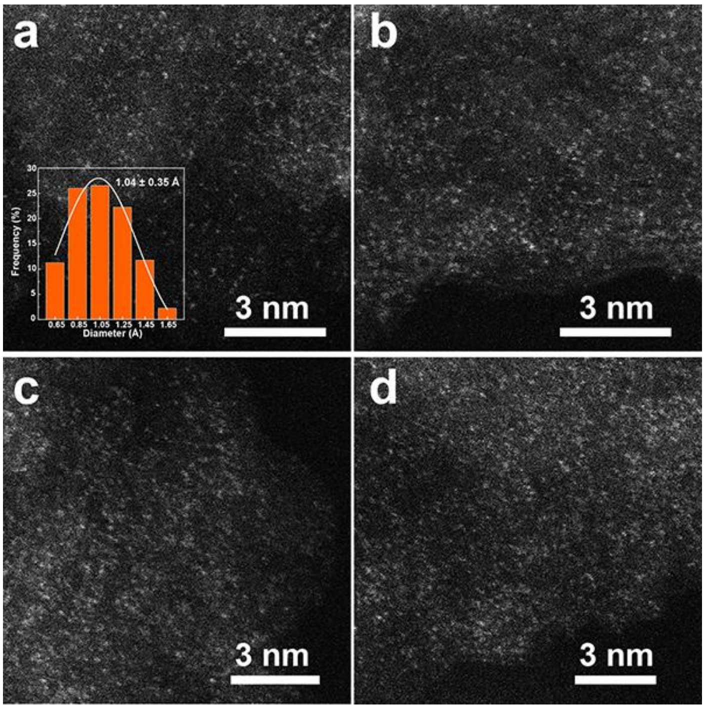

Supplementary Figure 8. a-d, Supplementary HAADF-STEM images of Fe-NC SAC. Inset in **a** is the size distribution of bright spots in HAADF-STEM images.

Comment 2: The authors have employed EXAFS to confirm the single atom nature of the products. They have compared the XAFS data to FePc structure. There are still contradictory reports on the EXAFS modeling of this type of compound. Furthermore, if we accept the model provided for FePc in this manuscript and assuming we have 8% Fe loading in the samples, why can't we see the second carbon shell similar to the FeC in the single atom samples' EXAFS spectra?

Response: Thanks for the comment. Since Fe atom is only bonded/coordinated with N atom in FePc, making it a clear reference to demonstrate the Fe-N structure, FePc was commonly used as the reference sample to evidence the formation of Fe-N structure in Fe-N-C materials reported in tremendous literatures, for example *J. Am. Chem. Soc. 139, 10790(2017) and Sci. Adv. 1, e1500462(2015).* We used the EXAFS data of our Fe-NC SAC to confirm that there is no Fe-Fe bond detected. Together with HAADF-STEM images and XRD results etc., this data supported the single atom nature of Fe-NC SAC. By comparing with the EXAFS and XPS data of FePc, Fe-NC SAC showed the feature of Fe-N configuration in terms of similar Fe-N distance and coordination number.

We think the main reason for "why our Fe-NC SAC did not show the clear signal of second carbon shell" is: unlike FePc which has clear molecular structure with second carbon shell, Fe-NC SAC was prepared by pyrolysis process, causing the second carbon

shells are not in good order. Such disorder can be observed in most of pyrolyzed Fe-N-C materials and carbon-based single atom catalysts (such as *Nat. Commun. 9, 3861(2018); Energy Environ. Sci. 11, 2348, (2018); Angew. Chem. Int. Ed. 55, 10800 (2016); Chem. Sci. 7, 5758 (2016)*). This disorder can also be evidenced by the wider Fe K-edge peak in XANES spectrum of Fe-NC SAC compared with FePc reference. The value of σ^2 (bond disorder) (7.9±1.0 for Fe-N) of Fe-NC SAC is significantly larger than the FePc reference (2.4±0.6 for Fe-N, 6.0±0.9 for Fe-C). The disorder of second carbon shell in Fe-NC SAC should be the main reason for its weak signal in the FT Fe K-edge EXAFS spectrum of Fe-NC SAC.

Comment 3: Comparing the wavelet transform of the Fe, FePc and the single atoms synthesized, it looks like the synthesized samples have a structure between Fe and FePc so claiming single atom formation based on this data is questionable.

Response: Thanks for the comment. As shown in Fig. 3c, WT analysis display only one intensity maximum at about 4.5 $Å^{-1}$ (4.3 $Å^{-1}$) for the Fe-NC SAC. Comparing with the intensity maximum at about 4.5 $Å^{-1}$ (4.6 $Å^{-1}$) for Fe-N bonding in the reference FePc and the one at 7.0 $Å^{-1}$ for Fe-Fe bonding in the reference Fe foil, it can be safely claim that this feature should be ascribed to Fe-N bonding, since it is very close to that of reference FePc and far from that for Fe-Fe bonding in the reference Fe foil.

It should be pointed out that the EXAFS data is used to support that no Fe-Fe bonding exists in our Fe-NC SAC. These data themselves cannot conclude the single atom nature. Together with HAADF-STEM images and XRD results etc., we could reasonably claim that Fe atoms are mainly in single-atomic state in Fe-NC SAC.

We have revised the discussion in the revision on Page 7 as follows:

As shown in **Fig.** 3c, WT analysis of Fe-NC SAC shows only one intensity maximum at about 4.5 $Å^{-1}$ for Fe-NC SAC, which is very close to that in the reference FePc (~4.5 $Å^{-1}$) but distinct from the feature of Fe foil (7.0 $Å^{-1}$).

Comment 4: Regarding the chemical state of Fe in the single atom products, the authors illustrate a +2 oxidation state. Can the authors comment on the oxidation state of other synthesized single atoms such as Pt using this synthesis process?

Response: Thanks for your comment. From the Fe 2p XPS spectrum, it can be clearly seen that Fe mainly exists in the form of Fe (II) and no clear signal for metallic Fe was

detected. As suggested, we further analyzed the chemical state of Pt, Ni, and Mn atoms in the corresponding catalysts by XPS technique. As shown in Figure R1, only characteristic peaks for Pt (II) (72.8, 76.1 eV) (*ACS Catal. 5, 2903 (2015); Nat. Commun. 8, 15938 (2017)*), Ni (II) (855.1, 873.5 eV) (*Angew. Chem. 54, 14031 (2015)*), and Mn (II) (641.9, 653.4 eV) (*J. Chem. Phys. 57, 973 (1972); Nat. Catal. 1, 935 (2018)*) are observed in the high-resolution Pt 4f, Ni 2p, and Mn 2p XPS spectra, respectively. These results suggest that the Pt, Ni, and Mn should also mainly exist in the form of Pt (II), Ni (II), and Mn (II) in the catalysts, respectively. More detailed analyses on the chemical state and bonding environment of metal atoms in M-NC SACs (besides the Fe-NC SAC) as well as various applications for other M-NC SACs are being carefully performed and will be included in the next publications.

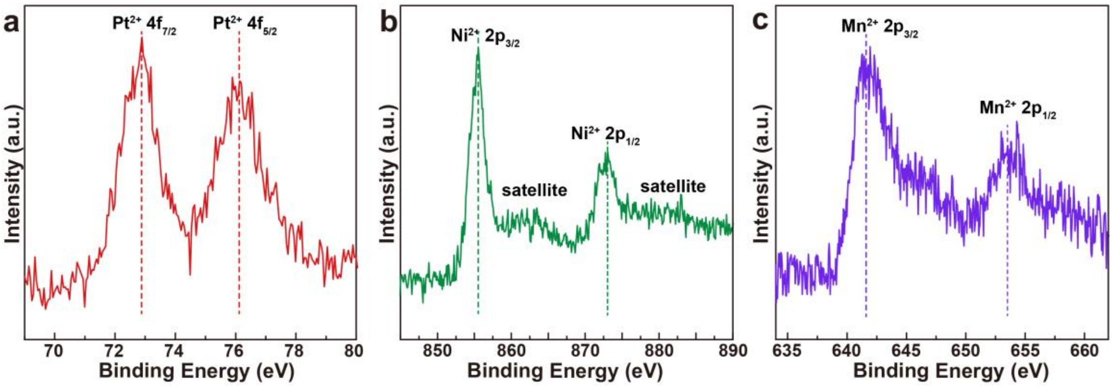

Fig. R1 a, Pt 4f XPS spectrum for Pt-NC SAC, **b,** Ni 2p XPS spectrum for Ni-NC SAC, and **c**, Mn 2p XPS spectrum for Mn-NC SAC.

Comment 5: The added text to the original manuscript require further polishing in terms of grammar and wording.

Response: Thank you very much for pointing out this issue. We have polished the manuscript accordingly.

Reviewer 2

I am happy with how the authors addressed most of the comments/concerns regarding the manuscript.

However, there are still 2 comments where I cannot find an agreement with the interpretation of the authors:

Comment 4: arguing that "a couple of spots shows the size slightly larger than single atom, which could be ascribed to the overlapping of atoms on the different depth profiles of carbon substrate or the rarely-seen atom cluster." is not a clear reflection of what the data show. using such a highly spatially resolved technique such as HAADF STEM imaging comes with the drawback that the depth resolution is quite limited. This means that if the atoms would be overlapping but on different "depth profiles" then they would need to be very close to each other-if they would be a few nm appart (z-direction) then this should lead to "blurring" and only one of the two imaged atoms would appear sharp.

I think the phrase "rarely seen atom clusters" needs to be removed as the data in Figure 6 clearly indicate the presence of a relatively high amount of clusters in at least b, e and f.

Response: Thank you very much for the helpful comments. In the previous manuscript, the sentence of "a couple of spots shows the size slightly larger than single atom, which could be ascribed to the overlapping of atoms on the different depth profiles of carbon substrate or the rarely-seen atom cluster" is not very accurate for descripting the HAADF STEM images. Basis on the HAADF STEM images (**Fig.** 2e and Supplementary **Fig.** 8), it can be seen that most of bright spots in each panel are actually in a size of single metal atom. We performed a statistical analysis on the size of these spots. Based on statistical analysis of over 400 bright spots, the average size of spots is 1.04 ± 0.35 Å. This result suggests that Fe atoms in the Fe-NC SACs should be mainly in single-dispersion state. Some of spots indeed displays slightly different diameters. We have discussed this result with the expert in this field of AC-STEM, Prof. Lin Gu at Institute of Physics, Chinese Academy of Sciences. This can be ascribed to the following two factors: 1) in view of the 3D structure of carbon support, the single metal atoms on different profiles were imaged at the same time. It is impossible to have all atoms at the same focusing plane, resulting in that the sizes of a few bright spots on HAADF-STEM images are slightly different; 2) Nowadays, it is still very difficult to

achieve or characterize 100 % single metal atom on bulk substrate in all reported methods. It cannot be 100 % sure in current stage that every atom is in individual state although the EXAFS data shows no metal-metal bonding in the sample. The similar phenomenon for TEM images can be observed in quite a lot of recent high-impact literatures about sing atom catalysts, such as *Science 352, 797(2016); Nat. Nanotechnol. 13, 411(2018); Sci. Adv. 1, e1500462(2015); Adv. Mater., 1705112(2018); Nat. Commun. 9,1460(2018) and Nat. Catal. 1, 781(2018)*. However, basis on our results, it should be convincible that an overwhelming majority of metal atoms in our report should be in single state.

As suggested, we have removed the phrase "rarely seen atom clusters" and revised the discussion in the revision on Page 14 as follows:

A couple of spots with different size could be ascribed to the metal atoms imaged at different focusing plane or possible atom cluster.

Comment 5: "the pixel size is set during the experiments and should not correlate to the atom size"
if you do not set the pixel size to the size of the atom but much bigger it is not possible to conclude that "N atoms are atomically adjacent to Fe atoms..."
I see that the text has been modified to "imply the possible existence of Fe-N bonding in the sample"; I want to stress again that the experimental EELS maps do not support this statement. Assuming your SI map has a pixel size of about 0.7 nm (scale bar is 2 nm and appears to be equivalent to about 3 pixels); this would mean an Fe and N atom could be 4 Angstrom apart and still be measured in the same "pixel".

Response: Thank you very much for the helpful comment. The pixel size indeed does not correlate to the atom size or the distance between Fe and N atom. As suggested, we have deleted the sentence of "imply the possible existence of Fe-N bonding in the sample".

REVIEWERS' COMMENTS:

Reviewer #1 (Remarks to the Author):

I like to thank you the authors for responding to all the questions and comments in detail.
I am satisfied with most of the answers and additional information provided.
I recommend this manuscript for publication without any further comments.

Reviewer #2 (Remarks to the Author):

Thank you for addressing the comments as requested.
